# Overexpression of *Lol-miR11467* negatively affects osmotic resistance in *Larix kaempferi* 3 × *L. gmelinii* 9

**DOI:** 10.1186/s12870-025-06591-x

**Published:** 2025-05-06

**Authors:** Sufang Zhang, Shanshan Yan, Li Zhang, Pingyu Yan, Hanguo Zhang, Lei Zhang

**Affiliations:** 1 State Key Laboratory of Tree Genetics and Breeding, Northeast Forestry University), Harbin, 150040 China; 2 Shandong Xiandai University, Jinan, 250000 China

**Keywords:** *Larix kaempferi* 3 × *L. gmelinii* 9, *Lol-miR11467*, Drought resistance, Transcriptome

## Abstract

**Background:**

Larch (*Larix gmelinii* (Rupr.) Kuzen.) is an important timber and ecological tree species in northern China. Excellent germplasm resources have been acquired through time-consuming traditional breeding. Molecular breeding offers a promising approach to shorten the breeding cycle and achieve genetic improvements more efficiently. MicroRNAs (miRNAs) are non-coding, single-stranded small RNAs that primarily affect plant growth and stress resistance, including drought stress. However, the study of miRNAs in larch under drought stress has not been well explored.

**Results:**

In this study, to investigate the function of *Lol-miR11467* under PEG osmotic stress in larch, embryogenic callus tissue of *Larix kaempferi* 3 × *L. gmelinii* 9 was employed as the experimental material, serving as the explants for this study. *Lol-miR11467* was transferred into the explants using an Agrobacterium-mediated method to determine the physiological changes and survey gene expression changes in overexpressing *Lol-miR11467* cell lines. The results showed that the fresh weight, peroxidase (POD), soluble protein and soluble sugar content of the overexpressing *Lol-miR11467* were lower than that of the wild-type, while malondialdehyde (MDA) content increased under PEG osmotic stress. Transcriptome analysis showed that genes associated with phenylpropanoid metabolism, transcription factors, oxidoreductase, plant hormone signal transduction, glucose metabolism and bioprotective macromolecules were mainly downregulated in *Lol-miR11467* cell lines.

**Conclusions:**

Overall, these results indicated that the drought resistance of the overexpressing *Lol-miR11467* cell lines was reduced. This study’s findings might provide a foundation for understanding the molecular mechanisms of miRNAs under PEG osmotic stress in larch, potentially contributing to the development of strategies for improving plant resilience to environmental stresses.

**Supplementary Information:**

The online version contains supplementary material available at 10.1186/s12870-025-06591-x.

## Background

*Larix* spp. belong to the *Pinaceae* of tall deciduous plants and are distributed in temperate mountainous and alpine climate regions of the Northern Hemisphere, primarily found in Northern China. Owing to its hard material, corrosion resistance, fast growth, strong stress resistance, and wide range of uses, it has become the main fast-growing, afforested, and economically important tree species in the northeast [1]. Decades of genetic improvement have achieved significant advancements in larch through hybrid breeding and seed orchard management, leading to the formation of many excellent families [2]. Nevertheless, challenges such as low seed yield, long fruiting cycles in seed orchards, combined with sexual reproduction, can lead to genetic segregation of excellent traits and considerable genetic variation. Traditional asexual reproduction methods, such as cutting and grafting, result in lower rooting rates and difficulties in large-scale production in the short term. Somatic embryogenesis and genetic engineering are expected to resolve these issues. Plant somatic embryogenesis involves the entire process of zygotic embryogenesis [3]. Transfer of beneficial exogenous genes into recipient larch cells, such as embryonic calli, through genetic engineering technology has emerged as a targeted genetic improvement strategy in plants. Currently, the time for molecular genetic improvement of larch is relatively short, and the genetic transformation system is not yet stable. Research on the molecular mechanisms far behind that of other tree species.

MiRNAs are a class of small non-coding RNAs that regulate target gene expression by directing transcript cleavage, translational repression, and histone modification at the translational or post-transcriptional levels [4–6]. They are involved in plant developmental processes, regulating the expression of transcription factors and physiological processes-related proteins. Therefore, miRNAs play a key role in plant growth, development, and responses to stresses [7]. MiRNAs are important regulatory factors involved in responses to drought and salt stress in plants [8]. Drought stress influences the expression of miRNAs in plants [9, 10]. MiRNAs have received much attention over the last two decades as major regulators of drought response in plants [11]. Research reports that miR169g was in the miR169 family of rice (*Oryza sativa* L.) under drought stress, and it is strongly upregulated under drought stress. Additionally, miR169 is expressed in tomatoes (*Solanum lycopersicum* L.), whereas miR169a and miR169c are inhibited in Arabidopsis [12, 13]. Although the miRNA sequences in the same family are relatively conserved, there may be functional differences among different plants. Studies have shown that the response of miRNAs to drought stress, exhibits species and tissue specificity. For example, miR168 and miR396 are expressed under drought stress conditions in Arabidopsis and tobacco (*Nicotiana tabacum* L.); however, their expression is inhibited in rice [14]. The expression of miR408 is inhibited by drought in rice but induced in Arabidopsis [15]. Different families also exhibit certain differences in expression under the same stress conditions. Under drought stress conditions, miR474 is expressed, whereas the expression of miR168, miR167, and miR582 is inhibited [16]. Research has shown that the overexpression of miRNAs can enhance or weaken plant drought resistance. For example, previous studies showed that the overexpression of tomato miR397 in Arabidopsis enhanced drought resistance [17]. Overexpression of miR169c in tomatoes and the downregulation of its target gene expression reduced transpiration rates in transgenic plants and decreased stomatal opening, indicating that miR169 responds to drought stress [18]. Overexpression of miR156 increases anthocyanin accumulation in *Medicago sativa*, thereby enhancing drought resistance [19]. Previous studies have indicated that members of the miR156 family participate in the response to drought stress in *Malus sieversii* [20], and msi-miR156 exhibits the same expression trend in both plant above-ground and below-ground. Overexpression of msi-miR156 in apple increased antioxidant enzyme activity and reduced reactive oxygen species (ROS), leading to enhanced abiotic stress resistance [21]. Due to frequent extreme weather conditions and severe spring drought in Northeast China, it seriously affects the survival and growth of larch. Therefore, it is necessary to study drought resistance in larch.

To study the regulatory function of miRNAs in response to PEG osmotic stress in larch, miRNAs were transferred into embryogenic calli using an optimized somatic embryogenesis and genetic transformation system [22]. Currently, research on the molecular mechanisms of larch is still in its early stages, and miRNAs that regulate downstream genes play an important regulatory role in this process [23]. At present, the miRNA information of conifers included in the miRBase database (https://www.mirbase.org) exists for only in four coniferous tree species: Chinese fir (*Cunninghamia lanceolata*), European spruce (*Picea abies*), red pine (*Pinus resinosa*), and loblolly pine (*Pinus taeda*). The miRNAs of larch have not been included, and the discovery and identification of miRNAs in larch are still in the early stages, which seriously affects the research progress on miRNAs in conifers. Fortunately, high-throughput sequencing of sRNA has addressed these challenges, with the sequencing read length of approximately 35 bp being suitable for sequencing short sequences of miRNAs. MiRNAs from European spruce (*Picea abies* (L.) H. Karst.) and loblolly pine (*Pinus taeda* L.) have also been isolated using sRNA library construction and sequencing [24]. This study was based on high-throughput sequencing of sRNA libraries to obtain miRNAs from larch under PEG osmotic stress. The *Lol-miR11467* sequence could be aligned with spruce trees, indicating that *Lol-miR11467* is likely to be a specific miRNA in conifers. To investigate the effect of overexpression of *Lol-miR11467* in larch on drought tolerance, *Lol-miR11467* was transferred into the embryogenic callus tissue of *Larix kaempferi* 3 × *L. gmelinii* 9 using the Agrobacterium-mediated method [22]. This analysis aimed to observe growth changes in overexpression of *Lol-miR11467* calli under PEG osmotic stress and to assess gene expression changes in the overexpression of *Lol-miR11467*. This study may provide a valuable basis for drought resistance mechanisms in larch.

## Results

### Construction of overexpression vector pCAMBIA1301-*Lol-miR11467*

The *Lol-miR11467* precursor amplification electropherogram showed a relatively bright band at the corresponding position (Figure S1a). After connecting the target gene *Lol-miR11467* to the vector pCAMBIA1301, *E. coli* colony PCR detection showed relatively bright bands at the target gene position, and the fragment size met the expectations (Figure S1b). The results indicated that the target gene, *Lol-miR11467*, was successfully cloned into the vector pCAMBIA1301. Six single colonies were selected and sequenced, and the alignment results indicated that the vector pCAMBIA1301-*Lol-miR11467* was successfully constructed. pCAMBIA1301-*Lol-miR11467* was transformed into Agrobacterium GV3101 and four single Agrobacterium colonies were selected. The PCR detection results showed relatively bright bands at the corresponding positions (Figure S1c), which were consistent with the expected results and could be used for infecting embryogenic callus tissues.

### Obtaining and detecting pCAMBIA1301-*Lol-miR11467* callus tissues

**Fig. 1:**
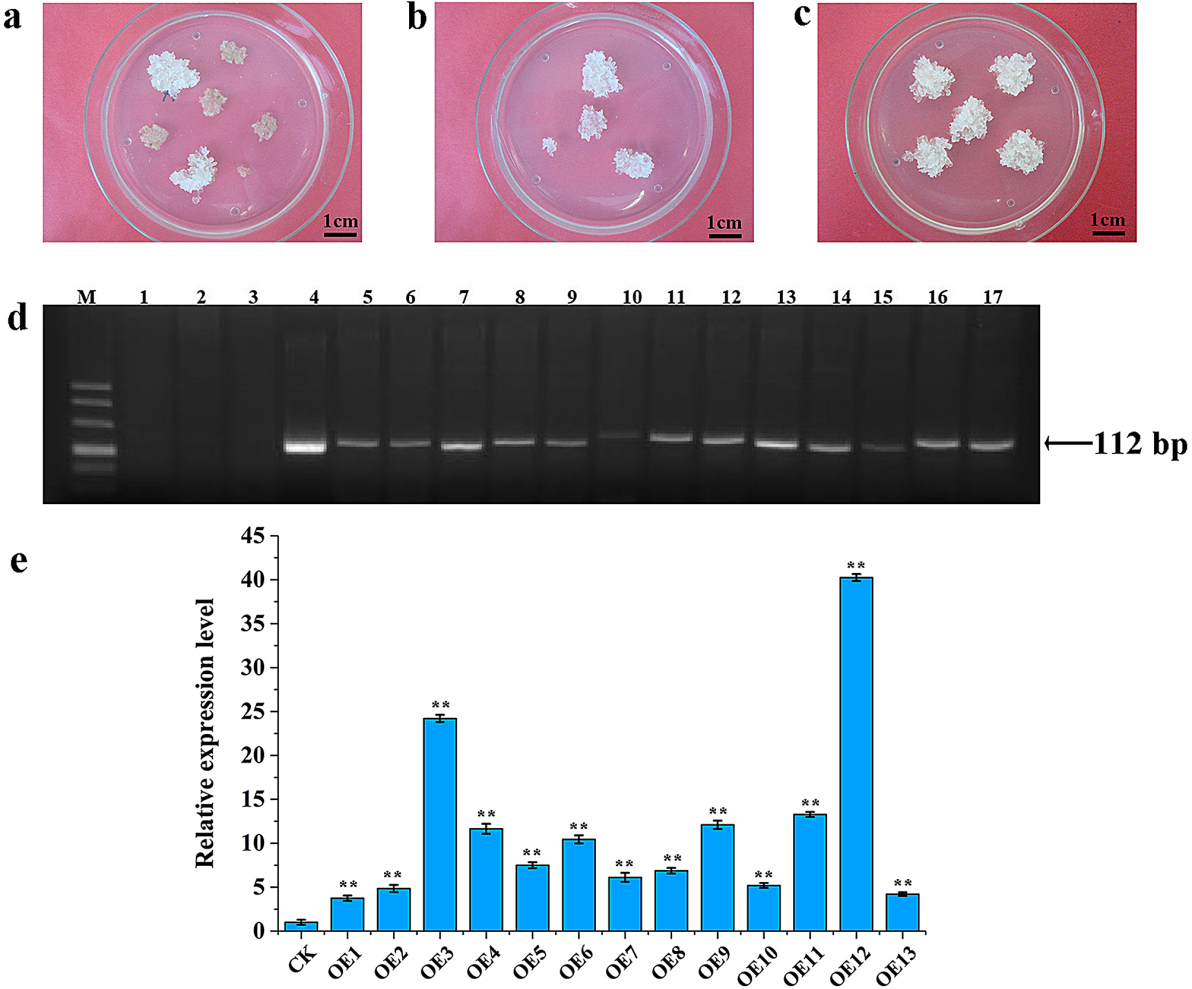
Obtaining and detection of pCAMBIA1301-*Lol-miR11467* callus tissues. **a**-**c**. The first, second and third round screening, respectively; **d**. PCR detection of transgenic embryogenic calli. M: Marker DL500; 1–3: PCR product of water, wild-type and empty vector; 4: PCR product of pCAMBIA1301-*Lol-miR11467* plasmid; 5–17: PCR product of pCAMBIA1301-*Lol-miR11467* embryonic callus cell lines; **e**. qRT-PCR detection of different transgenic embryogenic callus lines. CK is empty vector callus, OE1-OE13 are different transgenic calli. Three biology replicates are performed in the experiment. Data are showed with mean ± SD (*n* = 3). Differences are compared basing on t-test (* and ** indicate significant differences at the level of *p*<0.05 and *p*<0.01, respectively)

Embryonic callus tissues selected from the proliferation medium were used for genetic transformation of pCAMBIA1301-*Lol-miR11467*. After co-culturing for approximately two days, when Agrobacterium first appeared at the edge of callus tissues adjacent to the medium, it was subjected to sterilisation. After three rounds of surface cleaning with sterilised water, the sample was sterilised twice in a liquid medium containing 500 ppm cefotaxime for 5 min each time [22]. After culturing three rounds on the screening medium, resistant callus lines (callus tissues were grown in antibiotic screening medium) were obtained (Fig. 1a-c). PCR showed that the resistant calli had bands at the corresponding positions, indicating that *Lol-miR11467* was integrated into the genome of *Larix kaempferi* 3 × *L. gmelinii* 9 (Fig. 1d). Total RNA of 13 resistant calli and one empty vector-resistant callus was extracted and cDNAs were synthesised for qRT-PCR. The results showed that the relative expression levels of *Lol-miR11467* in all transgenic cell lines (OE1 - OE13) were higher than that in the CK (Fig. 1e), indicating that *Lol-miR11467* can be overexpressed normally. Among them, the expression levels in OE3 and OE12 were relatively high, which were 24.21 and 40.24 times higher than those of empty vector-resistant calli, respectively. Therefore, they were selected as experimental materials.

### Growth and morphological changes of Transgenic callus tissues under PEG osmotic stress

**Fig. 2:**
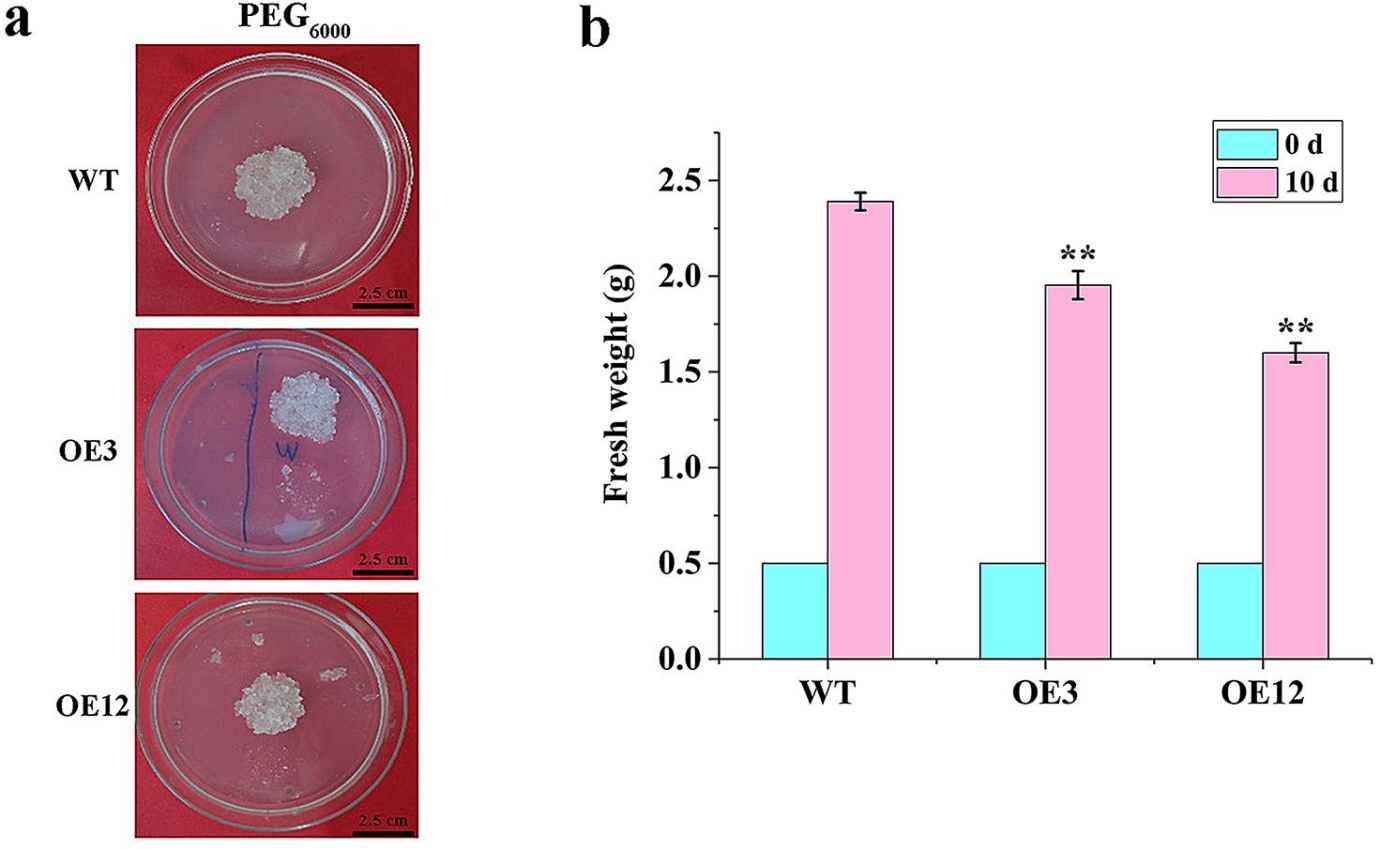
Growth status of calli under PEG_6000_ drought stress. **a**. The growth status of calli under PEG_6000_ drought stress after 10 days; **b**. The fresh weight of calli under PEG_6000_ drought stress after 10 days. Data are showed with means ± SD (*n* = 3). Differences are compared basing on t-test (* and ** indicate significant differences at the level of *p*<0.05 and *p*<0.01, respectively)

With the 10-day subculture cycle in mind, an investigation was carried out on callus tissues that had grown for 10 days [22]. Morphological and growth changes in the wild-type and transgenic calli after PEG_6000_ stress are shown in Fig. 2a. Compared to the wild-type, the fresh weight of transgenic calli continuously decreased with the increasing stress time (Fig. 2b). Notably, while the wild-type callus tissue exhibited slight browning, it displayed more proliferation than the transgenic callus tissue throughout the process (Fig. 2b).

In addition, under PEG_6000_ stress at different time points, the transgenic cell lines showed a sustained growth trend, which was more significant after stress 24 h and continued to grow until 96 h. The same pattern was observed in the wild-type, whereas the transgenic cell lines watered out more severely than the wild-type at 24 h (Figure S2).

### Determination of antioxidant enzyme activities of Transgenic callus tissue under PEG osmotic stress

**Fig. 3:**
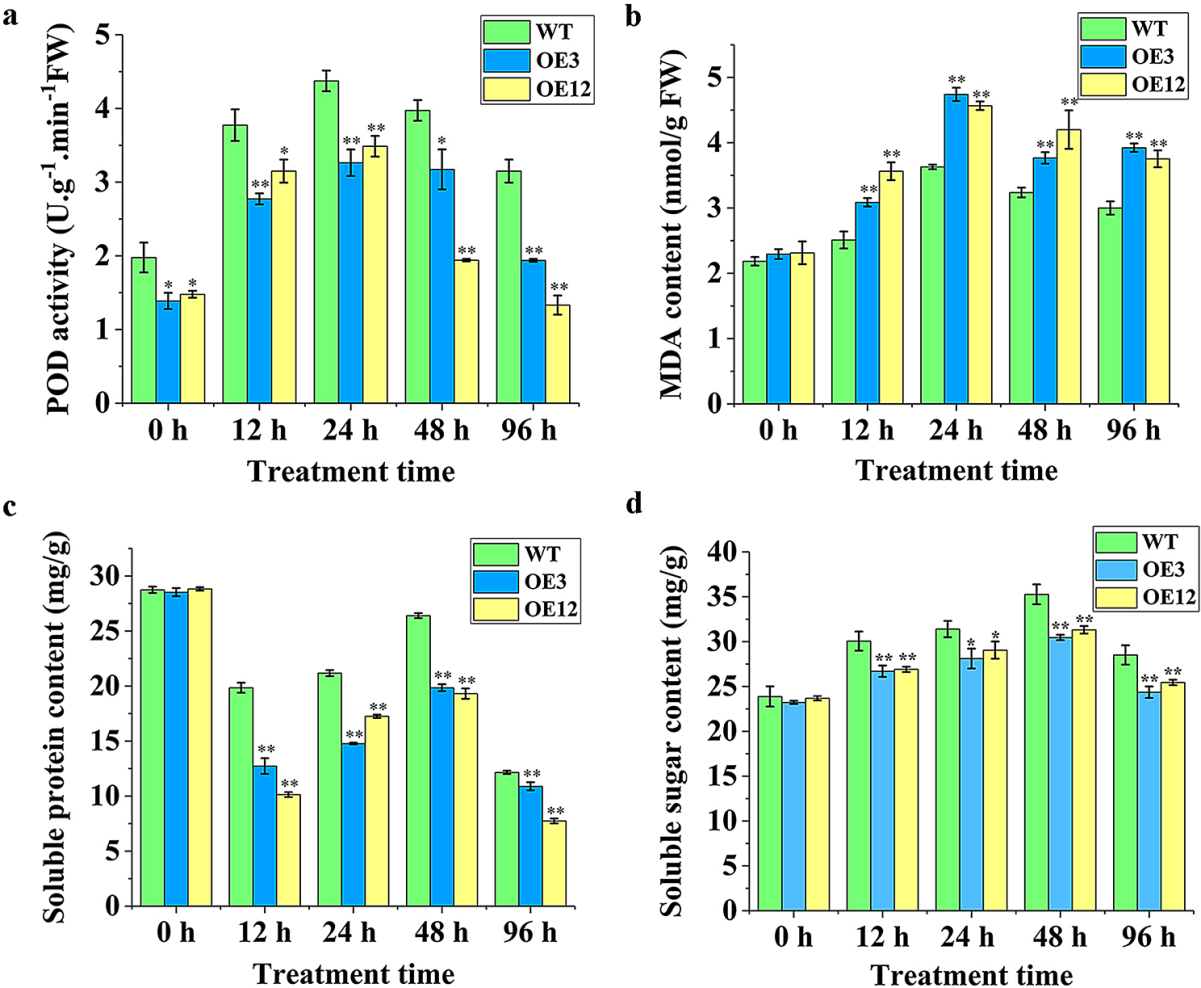
Determination of antioxidant enzyme activities of transgenic cell lines and wild-type under PEG osmotic stress. **a**. the activity of POD; **b**. the content of MDA; **c**. the content of soluble protein; **d**. the content of soluble sugar. Data are showed with means ± SD (*n* = 3). Differences are compared basing on t-test (* and ** indicate significant differences at the level of *p*<0.05 and *p*<0.01, respectively)

The activity of POD can serve as a reliable indicator of a plant’s ability to scavenge ROS, such as H_2_O_2_. The higher the POD activity, the stronger the plant physiological metabolism and antioxidant capacity, which can accelerate the clearance of reactive oxygen and enable better adaptation to stress conditions [25]. Under PEG_6000_ stress, the activity of POD in both transgenic cell lines OE3 and OE12 and wild-type first increased and then decreased, reaching its peak at 24 h. Despite an overall decline in enzyme activity with the increase of stress time, the activity of POD in OE3 and OE12 was lower than of the wild-type. Although *Lol-miR11467* can regulate cell lines in response to PEG osmotic stress, the osmotic resistance of OE3 and OE12 may be weakened (Fig. 3a). The amount of MDA can serve as an indicator of the extent of oxidative damage caused by stress in plants. Meanwhile, POD can effectively eliminate excessive ROS in a timely manner and remove excess MDA [26]. Under PEG_6000_ stress, the content of MDA in both the wild-type and transgenic cell lines mostly first increased and then decreased with an increase in stress time, and reached its maximum at 24 h. Furthermore, the content of MDA in OE3 and OE12 was higher than that in the wild-type at different time, suggesting that a greater accumulation of harmful substances in the OE3 and OE12. This indicated that the transgenic cell lines were more susceptible to oxidative damage than the wild-type cells, leading to weakened drought resistance (Fig. 3b). In summary, it is evident that under PEG_6000_ stress, the content of MDA in OE3 and OE12 was consistently higher than that in the wild-type followin*g* the start of stress. This indicates that the transgenic cell lines undergo peroxidation reactions after being subjected to PEG osmotic stress, resulting in the accumulation of higher MDA levels and increased damage compared to the wild-type. Meanwhile, the content of soluble protein in both transgenic cell lines and wild-type first decreaed, then increased, and finally decreased again with an increase in stress time. After the start of stress, the soluble protein content decreased compared with that without stress. Compared with POD enzyme activity and MDA content, the range of changes in soluble proteins was larger and decreased after 12 h of PEG_6000_ stress. The soluble protein content increased again after 24–48 h of stress (Fig. 3c), possibly because of the induction of proteins translation, such as stress proteins. It began to decrease again after 96 h, possibly due to the breakdown of soluble proteins into amino acids, which reduced the osmotic potential of the cell lines and promoted water absorption. Besides, the soluble sugar content gradually increased within 24 h as the stress time is prolonged, decreased after 48 h of stress, increased again after 96 h of stress (Fig. 3d). Therefore, it can be speculated that, in the short term, soluble sugars in larch callus tissue act as the important osmotic regulators to reduce cell osmotic potential and enable the tissue to adapt to unfavorable environmental conditions. Overall, the content of soluble protein and soluble sugar in OE3 and OE12 was lower than in the wild type at different stress time points, indicating that overexpression of *Lol-miR11467* may regulate negatively the soluble protein content of transgenic cell lines, reduce their resistance to abiotic stress, and thus exhibit lower drought resistance than the wild type (Fig. 3c, d).

### Identification of differentially expressed genes (DEGs)

**Fig. 4:**
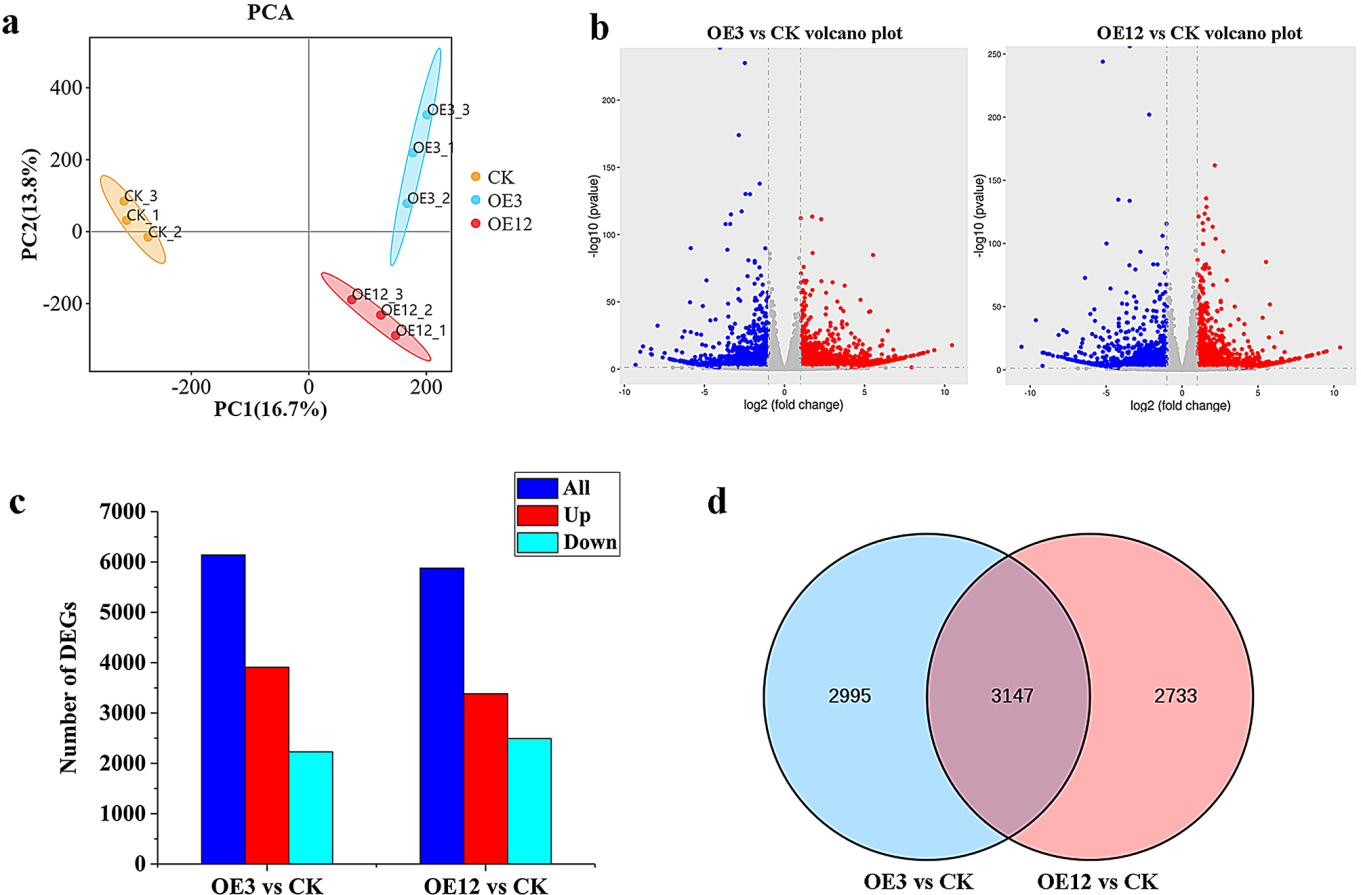
Analysis of DEGs in transgenic cell lines. **a**. PCA diagram of DEGs; **b**. The Volcanic map of DEGs in OE3 vs. CK and OE12 vs. CK; **c**.The number of DEGs in OE3 vs. CK and OE12 vs. CK, the navy blue bar represents all DEGs, up-regulated genes are in red, and down-regulated genes are in light blue, log2 (Fold change)|>1 and *p*-value < 0.05. **d**. The Venn diagram of DEGs in OE3 vs. CK and OE12 vs. CK

To further analyse the regulatory function of *Lol-miR11467* in larch, the function of annotated DEG and the metabolic pathways involved were analyzed. The empty vector pCAMBIA1301 resistant callus CK and resistant callus cell lines OE3 and OE12, which exhibited higher expression levels than CK, were selected for transcriptome sequencing analysis. After filtering out short and low-quality reads, a total of 549,354,430 clean reads were obtained, ranging from 3.8G to 5.3G. The Q30 was 92.04-94.36%, and the GC content was 43.69-44.47% (Table S7). Generally, mapped read rates greater than 70% are considered no pollution and the reference genome is appropriate [27]. In this study, the mapped read rates of the six samples were 74.31% (74.53%, 74.63%), 74.46% (74.39%, 74.49%), and 74.35% (74.77%, 73.90%), all greater than 70% (Table S8), indicating that the reference genome was appropriate. Moreover, the transcripts were clustered into three groups by principal component analysis (PCA) based on transgenic cell lines and CK, indicating there were differences and good correlations among the samples (Fig. 4a). These results indicated that the RNA-seq was of good quality and coul*d* be used for further analyses.

The DEGs results showed that compared with CK, the DEGs in the OE3 and OE12 cell lines were relatively close, and there were more upregulated DEGs in each cell line (Fig. 4b-c). Generally, miRNAs negatively regulate their target genes; thus, *Lol-miR11467* may negatively regulate the mentioned above upregulated DEGs during the growth and development of larch. The Venn diagram of DEGs in each cell line showed that there were 3147 common DEGs between OE3 and OE12, 2995 DEGs specific to OE3, and 2733 DEGs specific to OE12 (Fig. 4d). Because both OE3 and OE12 are overexpressing *Lol-miR11467* cell lines, the common 3147 DEGs were selected for subsequent analysis.

### Verification of RNA-seq data

**Fig. 5:**
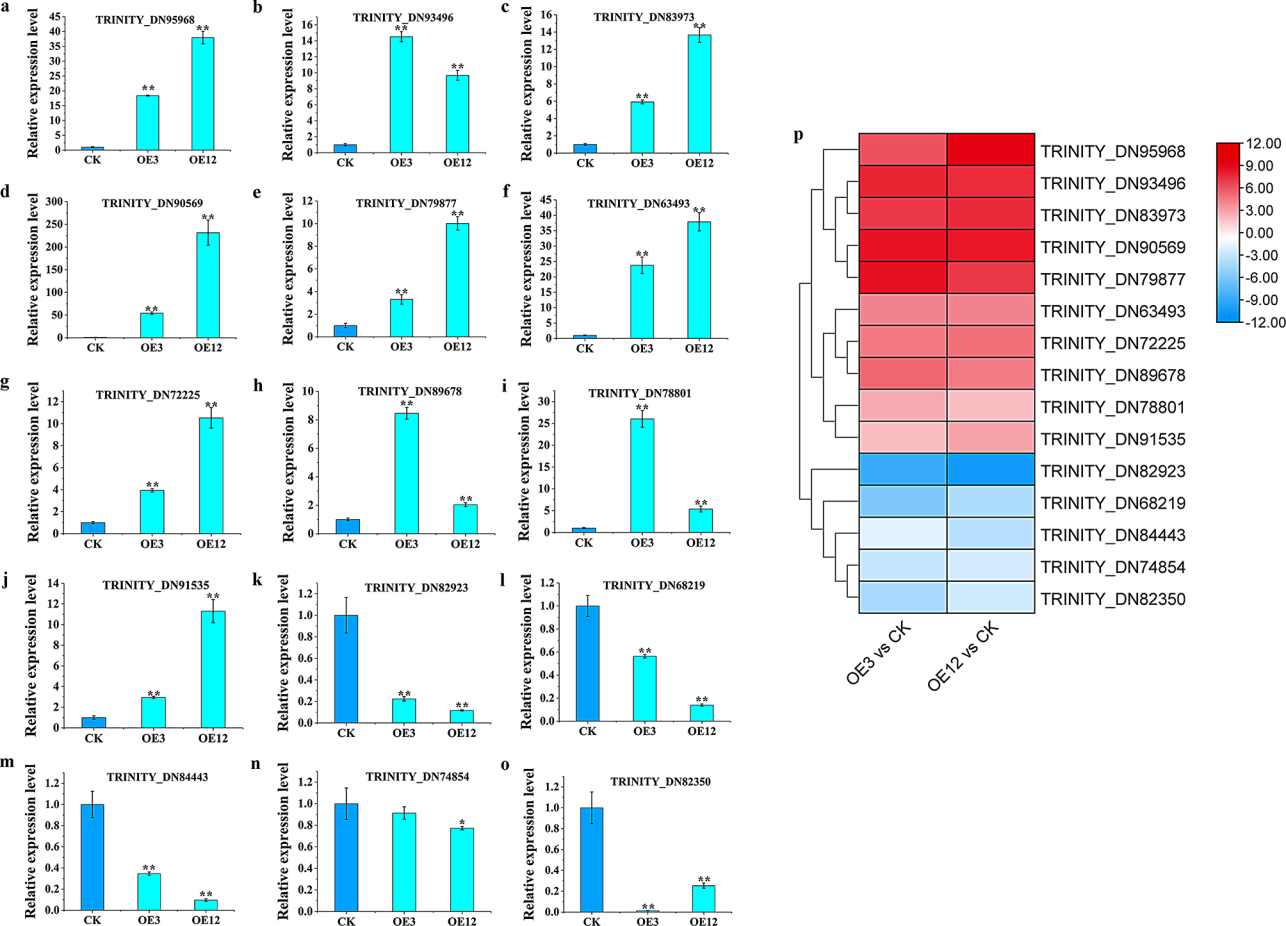
Verification of RNA-seq data. **a**-**o**: Data are showed with means ± SD (*n* = 3). Differences are compared basing on t-test (* and ** indicate significant differences at the level of *p*<0.05 and *p*<0.01, respectively). The expression level of each gene in the CK was “1”. The qRT-PCR primers were listed in Table S6; p: RNA-Seq data of fifteen DEGs

RNA-seq data showed that many DEGs were significantly upregulated or downregulated. Fifteen DEGs were selected for verifing the reliability of RNA-seq. The results indicated that although the expression levles of 15 genes in qRT-PCR (Fig. 5a-o) and RNA-seq (Fig. 5p) were different, which may be due to the sensitivity difference between these two detection methods, their expression trends were consistent. Therefore, the RNA-seq data were reliable and could be used for subsequent gene function analysis. In addition, when exogenous genes are introduced were into plant cells, they are integrated into the nuclear genome through the replication process of genetic material during cell division. Therefore, the expression of some genes were different in both OE3 and OE12 cell lines, possibly due to the different insertion sites of *Lol-miR11467* on the chromosome of larch.

### Cluster expression and annotation analysis of DEGs

**Fig. 6:**
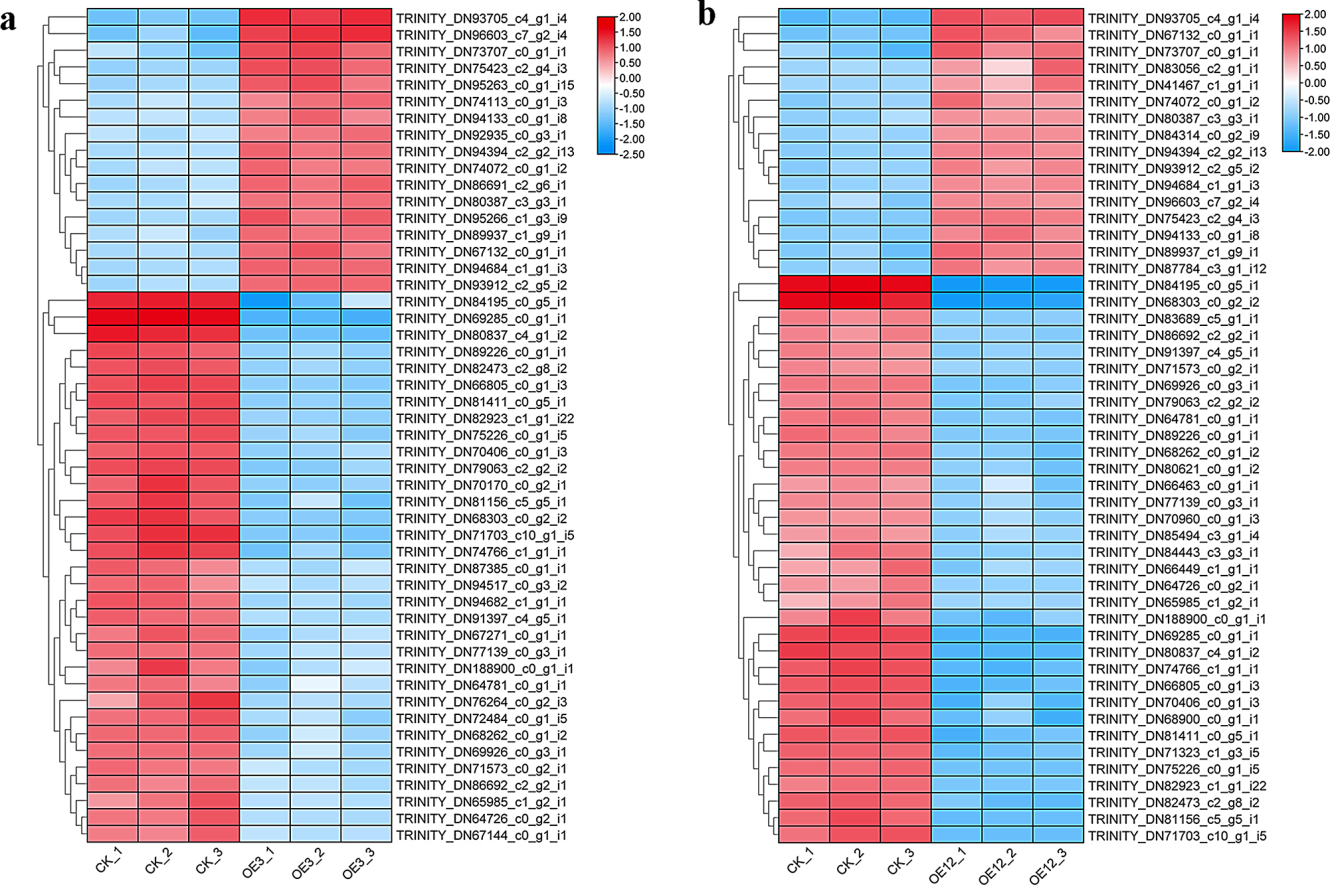
Heatmap of DEGs in the top 50 of OE3 and OE12 transgenic cell lines. CK_1, CK_2, and CK_3 represnt three replicates of control; OE3_1, OE3_2, and OE3_3 represnt three replicates of transgenic cell line OE3; OE12_1, OE12_2, and OE12_3 represnt three replicates of transgenic cell line OE12. **a**. Heat map of DEGs in the top 50 of OE3; **b**. Heat map of DEGs in the top 50 of OE12. *p*-value < 0.05 and (|log2FC = > 1) are used to screen for DEGs

To compare the expression clustering patterns of DEGs in OE3 and OE12 with those of the CK, clustering heatmaps of DEGs were drawn for the top 50 with the highest variance in expression levels in OE3 vs. CK and OE12 vs. CK (Fig. 6). The results showed that there were 33 and 34 downregulated DEGs in OE3 vs. CK and OE12 vs. CK, respectively, accounting for a large proportion. This suggested that the overexpression of *Lol-miR11467* had a negative regulatory effect on its target genes and disrupted the normal transcription level of larch. The heatmap also showed that the three biological replicates were consistent in each sample. Interestingly, some genes (such as TRINITY_DN96603_c7_g2_i4, TRINITY_DN75423_c2_g4_i3, TRINITY_DN94113_c0_g1_i8, etc.) expressed in different pattern in both OE3 and OE12 lines. The reasons may include the introduction of exogenous genes into plant cells, which can result in different insertion sites on the chromosomes. Additionally, the locations where integrations occur within the host chromosome are random, meaning they can be inserted into any chromosome of the plant’s genome, at any position on a chromosome, or even without a specific insertion site. In addition, these annotated DEGs are listed in Table S9. The results indicated that most annotated in conifers, such as *Picea sitchensis*, had unknown functions, while encoding elongation factor 1-alpha isoform X1, AT-hook motif nuclear-localised protein, SCF ubiquitin ligase, nuclear transport factor 2 family protein, and many hypothetical proteins were annotated to other species.

### Functional enrichment analysis of DEGs

**Fig. 7:**
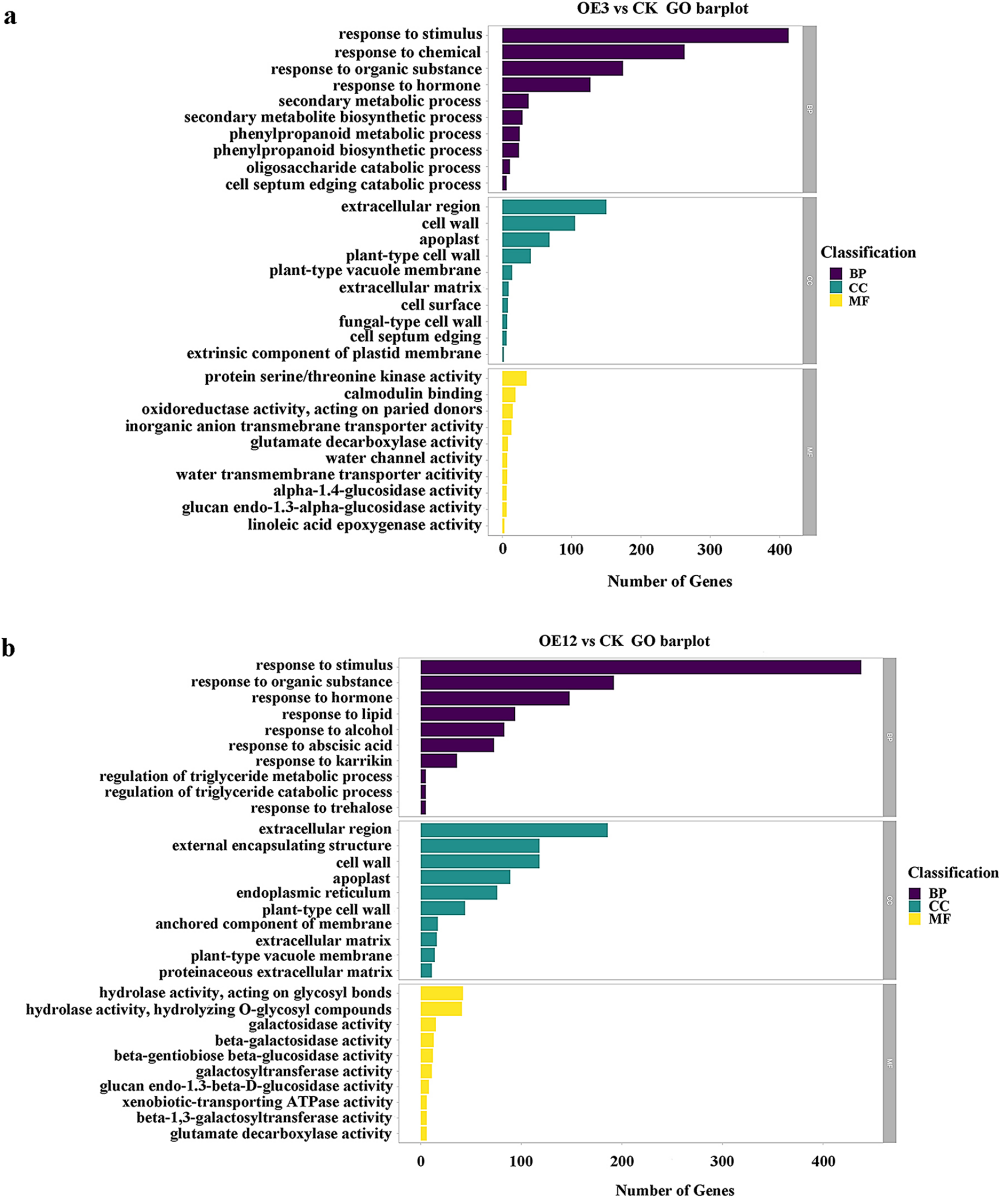
GO enrichment analysis of DEGs in OE3 vs. CK and OE12 vs. CK. **a**. GO enrichment analysis of OE3 vs. CK; **b**. GO enrichment analysis of OE3 vs. CK. The abscissa represents GO term. The significance threshold is *p*-value < 0.05

To further survey the function of DEGs in overexpressing *Lol-miR11467*, GO annotation and enrichment analyses were conducted on DEGs, which were annotated into the three branches of cellular component (CC), biological process (BP), and molecular function (MF) in GO classification. Among the GO terms (Table S10), response to hormone (GO:0009725), glucan endo-1,3-alpha-glucosidase activity (GO:0051118), and extracellular region (GO:0005576) were significantly enriched in both the OE3 vs. CK and OE12 vs. CK groups. Furthermore, the top 10 branches of GO were ranked (Fig. 7a, b). We compared OE3 vs. CK and OE12 vs. CK groups (Fig. 7). The results showed that in BP classification, the GO terms were mainly enriched in response to stimulus (GO:0050896), cell septum edging catabolic process (GO:0030995), phenylpropanoid biosynthetic process (GO:0009699), etc., in the OE3 vs. CK groups, while response to hormone (GO:0009725), response to organic substance (GO:0010033), etc., were mainly enriched in the OE12 vs. CK group. In CC classification, the cell wall (GO:0005618) apoplast (GO:0048046), and extracellular region (GO:0005576) in both OE3 vs. CK and OE12 vs. CK groups were mainly enriched. In MF classification, glutamate decarboxylase activity (GO:0004351), glucan endo-1,3-alpha-glucosidase activity (GO:0051118) and calmodulin binding (GO:0005516) were significantly enriched in the OE3 vs. CK group, while glucan endo-1,3-beta-D-glucosidase activity (GO:0042973), beta-gentiobiose beta-glucosidase activity (GO:0080083) and beta-galactosidase activity (GO:0004565) were significantly enriched in the OE12 vs. CK group.

**Fig. 8:**
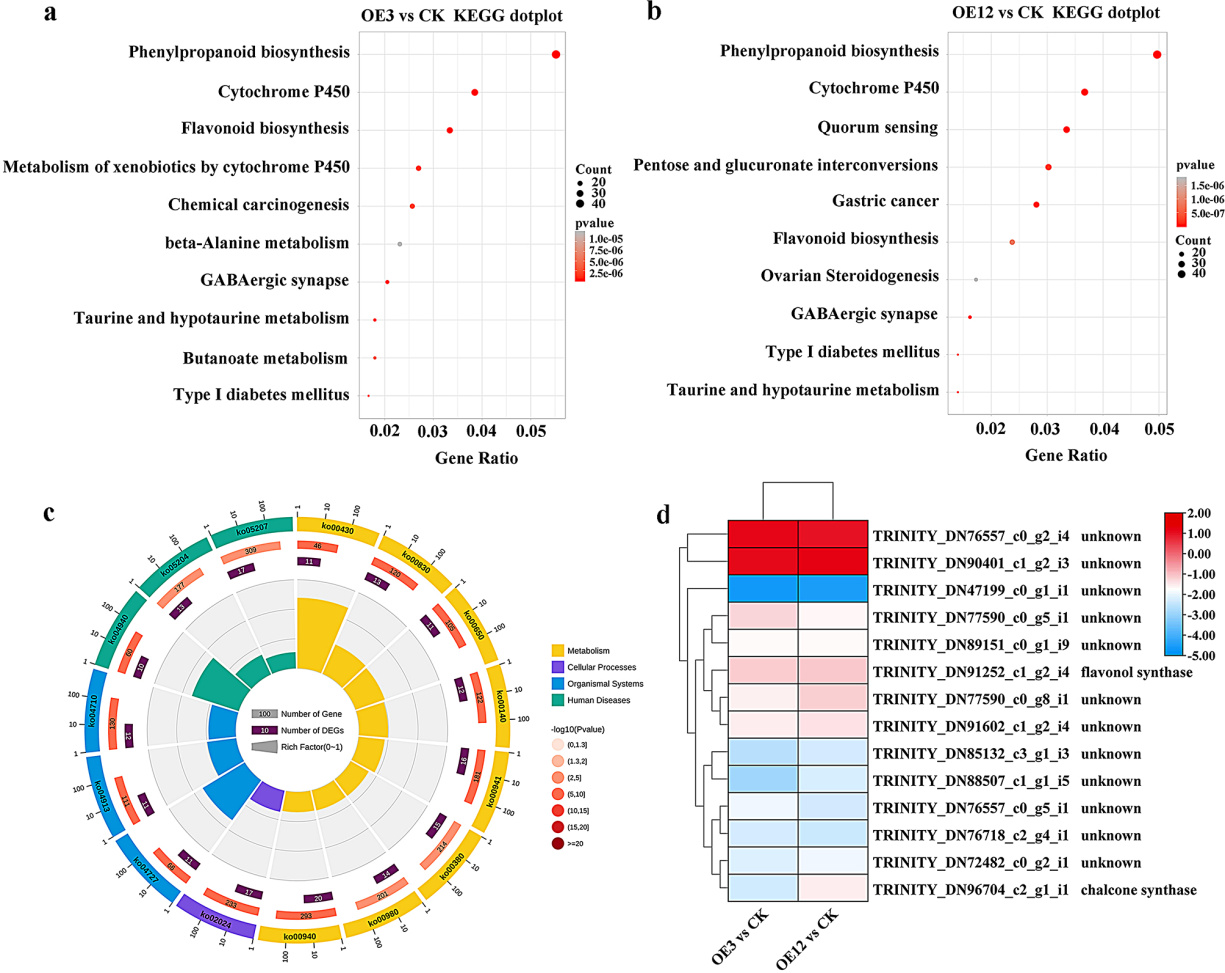
KEGG enrichment analysis of DEGs in OE3 vs. CK and OE12 vs. CK. **a**. The KEGG enrichment of DEGs in OE3 vs. CK; **b**. The KEGG enrichment of DEGs in OE12 vs. CK; **c**. The KEGG enrichment of common DEGs in OE12 vs. CK and OE12 vs. CK; **d**. The common DEGs in flavonoid biosynthesis pathway. The significance threshold is *p*-value < 0.05

Furthermore, KEGG pathway annotation and enrichment analysis were performed. In the OE3 vs. CK group, a total of 2553 DEGs were annotated and enriched in 356 metabolic pathways (Table S11). The most significantly enriched DEGs were involved in phenylpropanoid biosynthesis (ko00940), followed by cytochrome P450 (ko00199), flavonoids biosynthesis (ko00941), xenobiotic metabolism by cytochrome P450 (ko00980), and beta-alanine metabolism (ko00410) (Fig. 8a). In the OE12 vs. CK group, a total of 3276 DEGs were annotated and enriched in 350 metabolic pathways (Table S11). The most significantly enriched DEGs were involved in phenylpropanoid biosynthesis, followed by the cytochrome P450 and flavonoid biosynthesis pathways (Fig. 8b). Based on the above results, phenylpropanoid biosynthesis, cytochrome P450, and flavonoid biosynthesis were enriched in both OE3 vs. CK and OE12 vs. CK; phenylpropanoid biosynthesis accounted for a significant proportion in both groups, and its enrichment was also the most significant (Fig. 8a, b). As both OE3 and OE12 were overexpressing *Lol-miR11467* cell lines, the common 3147 DEGs were selected for KEGG enrichment analysis. It was also found that the most significant and annotated DEGs were in the phenylpropanoid metabolism pathway (Fig. 8c). These results suggested that *Lol-miR11467* played a key role in the metabolic pathway of phenylpropanoid biosynthesis in larch and had a regulatory effect on its growth and development. Notably, flavonoid biosynthesis was observed in both OE3 vs. CK and OE12 vs. CK, and the upregulated genes related to flavonoid synthesis enhanced drought resistance [28]. In this study, the flavonoid biosynthesis pathway was significantly enriched, and most related genes were downregulated (Fig. 8c, d). Therefore, it is speculated that the drought resistance of the overexpressing *Lol-miR11467* cell lines might be weakened.

### Genes involved in drought response were down-regulated in Transgenic cell lines

**Fig. 9:**
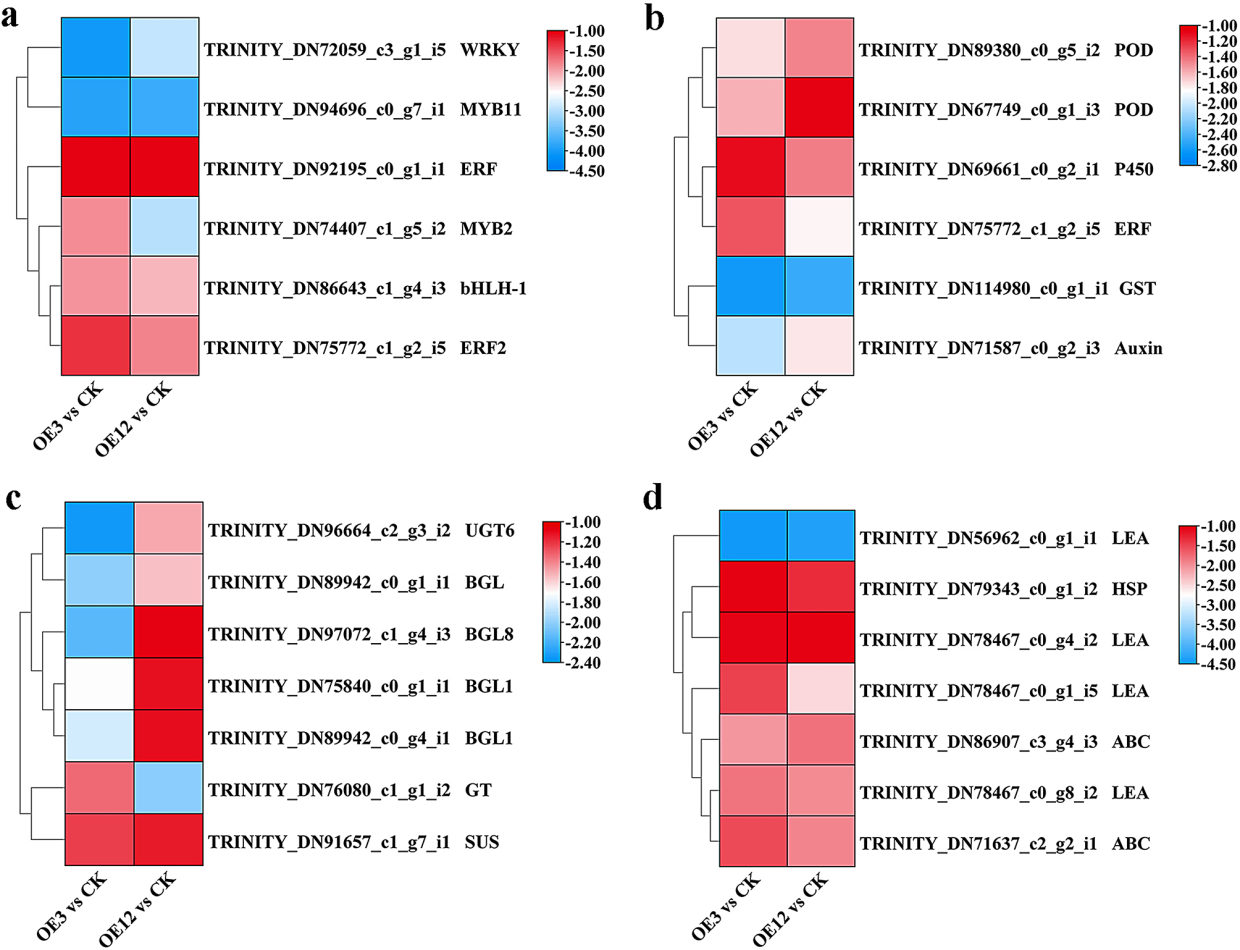
Downregulated DEGs of OE3 vs. CK and OE12 vs. CK. **a**. Transcription factors genes; **b**. Hormone-related genes and ROS-related genes; **c**. Glucose metabolism-related genes; **d**. Functional protein-related genes. Heatmap of columns and rows represent samples and genes, respectively. POD: peroxidase; P450: cytochrome P450; ERF: ethylene-responsive transcription factor; GST: glutathione S-transferase; UGT: UDP-glycosyltransferase; BGL: beta-galactosidase; GT: glycosyltransferase; SUS: sucrose synthase; LEA: late embryogenesis abundant; HSP: Heat shock protein; ABC: ABC transporter

Given that both OE3 and OE12 are overexpressing *Lol-miR11467* cell lines, and miRNAs generally negatively regulate their target genes, it is important to further determine which DEGs are involved in the regulation. Therefore, the downregulated genes among the 3147 common DEGs were analysed. Many studies have shown that transcription factors, which are trans-acting factors that directly regulate gene expression, play important roles in plant stress [29]. Studies have also shown that transcription factors related to drought resistance mainly include the DREB, bZIP, WRKY, MYB/MYC, and NAC5 families. Overexpression of these factors activates many stress-resistant functional genes and coordinates their expression, thereby improving plant drought resistance and vice versa [30]. Therefore, analysing transcription factors related to drought can also provide important information on the complex regulatory network of larch under PEG osmotic stress. In this study, the WRKY, MYB, ERF, and bHLH transcription factors were downregulated in both OE3 and OE12 cells (Fig. 9a), which may have an impact on the growth and development of transgenic cell lines.

Studies have shown that many miRNAs respond to drought stress by regulating the expression of transcription factors, genes involved in antioxidant defence mechanisms, and signal transduction. Changes in transcription factor genes can induce the expression of downstream genes that can alter plant stress resistance. In the present study, DEGs related to oxidoreductases and plant hormone signal transduction, such as POD, P450, GST, ERF, and AUXIN, were found to be downregulated (Fig. 9b). Therefore, the downregulation of these genes may inhibit the ability of transgenic cell lines to resist stress.

Some studies have shown that plants can resist drought by accumulating osmotic regulatory substances such as soluble sugars and proteins, which help maintain plant growth normally under drought stress [31, 32]. UDP-glucosyltransferase (UGT) is essential for the growth and development of many plants and plays a crucial role in abiotic stress adaptation [33]. Galactosidases (BGL) belong to a relatively wide glycosyl hydrolases family found in plants that participate in the metabolic processes of galactosides, glycoproteins, and cell wall polysaccharides. These enzymes break down polysaccharides into oligosaccharides, increasing the osmotic potential of plant cells and enhancing drought resistance [34]. In this study, common DEGs in both OE3 and OE12 included the above enzyme-related genes, such as UDP-glycosyltransferase (*UGT*), beta-galactosidase (*BGL*), glycosyl transferase (*GT*) and sucrose synthase (*SUS*) (Fig. 9c). Therefore, overexpression of *Lol-miR11467* can lead to the downregulate of genes related to drought resistance, suggesting reduced drought resistance in transgenic cell lines.

In this study, these DEGs were downregulated, including *LEA* (late embryogenesis-abundant), *HSP* (heat shock protein), and *ABC* (ATP-binding cassette) (Fig. 9d). LEA and HSP are associated with stress, indicating that the overexpression of *Lol-miR11467* can negatively regulate the expression of these genes, thereby potentially weakening the stress resistance of transgenic cell lines.

**Fig. 10:**
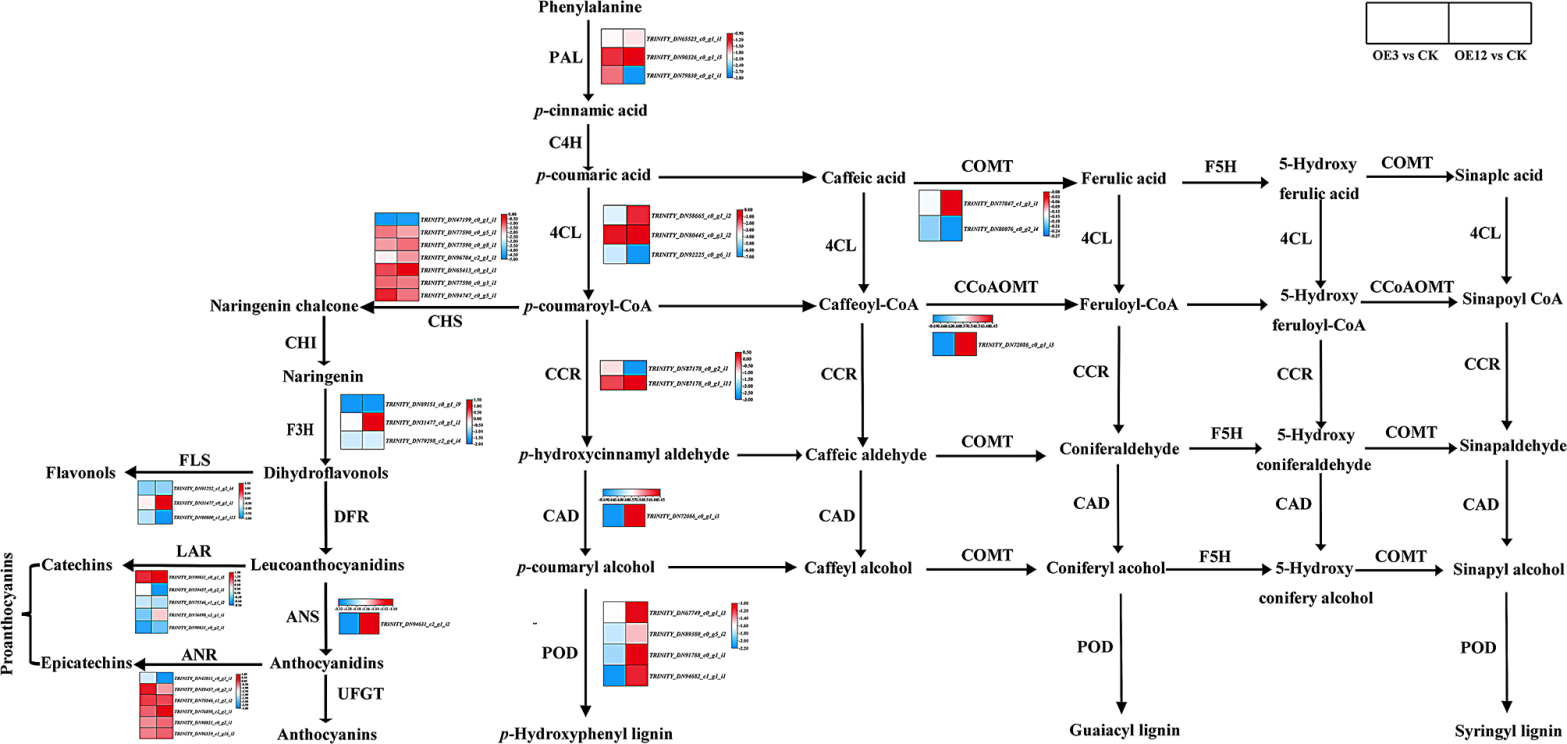
DEGs related to phenylalanine biosynthesis were mainly down-regulated in both the OE3 vs. CK and OE12 vs. CK. A simplified biosynthetic pathway representing the most common pathways of the three major lignin units and flavonoid biosynthesis. Heatmap of columns and rows represent samples and genes, respectively. PAL: phenylalanine ammonia lyase; C4H: coumarate-4-hydroxylase; 4CL: 4-coumarate CoA Ligase; COMT: caffeic acid O-methyltransferase; CCoAOMT: caffeoyl CoA O-methyltransferase; CCR: cinnamoyl-CoA reductase; CAD: cinnamyl alcohol dehydrogenase; POD: peroxidase; CHS: chalcone synthase; CHI: chalcone isomerase; F3H: Flavanone 3-hydroxylase; FLS: flavonol synthase; DFR: dihydroflavonol reductase; LAR: leucoanthocyanidin reductase; ANS: anthocyanidin synthase; ANR: anthocyanidin reductase; UFGT: UDP-glucoside: flavonoid glucosyltransferase

Based on KEGG pathway analysis, the phenylalanine biosynthesis process were significantly enriched in both the OE3 vs. CK and OE12 vs. CK (Fig. 10). Further analysis showed that lignin biosynthesis genes *PAL*, *4CL*, *COMT*, *CCoAOMT*, *CAD*, *CCR*, and *POD* were downregulated in both the OE3 vs. CK and OE12 vs. CK. Additionaly, the transcription levels of *CHS*, *F3H*, *FLS*, *LAR*, *ANS* and *ANR* in the flavonoid biosynthesis pathway were mainly downregulated in the OE3 vs. CK and OE12 vs. CK.

### Prediction of *Lol-miR11467* target genes

Based on the specific targeted binding relationship between miRNA and its target genes, the sequences of DEGs from resistance callus tissue transcriptome were used as reference sequences and aligned with the mature sequence of *Lol-miR11467* for target gene prediction. Sequence alignment was conducted using miranda v3.3a software (microRNA Target Scanning Algorithm) developed by the Memorial Sloan Kettering Research Center. Four potential target genes that *Lol-miR11467* may act on include: *TRINITY_DN78358_c2_g2_i1*, *TRINITY_DN76273_c0_g3_i5*, *TRINITY_DN87896_c2_g1_i9*, and *TRINITY_DN95853_c3_g3_i1* (Table S12). To further investigate whether *Lol-miR11467* acts on its predicted target genes, we performed qRT-PCR analysis on the target genes in transgenic cell lines OE3 and OE12, the primers for qRT-PCR was listed in Table S13. The results showed that compared with the wild-type, the expression levels of the four genes significantly decreased in both OE3 and OE12 lines (Figure S3). Therefore, *Lol-miR11467* may acts on its four predicted target genes by negatively regulating *Lol-miR11467*.

## Discussion

### Genetic transformation of Lol-miR11467 in Larix kaempferi 3 × L. gmelinii 9

Transgenic overexpression is a commonly used method for studying gene function, and it can be used functional studies of miRNAs. Overexpression of miR171a significantly enhanced drought tolerance in rice [35]. Overexpression of miR390b in poplar (*Populus* L.) promoted stem elongation and plant height [36]. Additionally, the overexpression of miR390b in rice was found to enhance the oxidative stress resistant. Similarly, overexpressing miR529a in *Oryza sativa* enhanced resistance to oxidative stress. Overexpressing miR156e or miR529a resulted in increased grain size and tiller, but decreased plant height and panicle length [37]. Conversely, the opposite phenotype was observed in their target mimicry transgenic plants [38]. Transgenic plants have shown a higher antioxidant capacity under drought stress [39]. In this study, the resistant cell lines (Fig. 1) by overexpressing *Lol-miR11467* in *Larix kaempferi* 3 × *L. gmelinii* 9 were obtained. The co-culture time is crucial during genetic transformation, and the time too long or too short can affect later sterilisation, leading to incomplete sterilisation and callus tissues death. In addition, after sterilisation, it is crucial to remove as much water as possible from the surface of the callus tissues to achieve better sterilisation. Generally, the co-culture time is 2–3 days, but in practical experiments, the optimal time should also be adjusted based on different strains or concentration of bacterium, mainly based on the growth state of *Agrobacterium tumefaciens* at the edge of callus tissues. When the co-culture time is too long, the toxicity of Agrobacterium to callus tissue can be reduced by increasing the sterilisation time.

### The drought resistance of overexpressing *Lol-miR11467* cell lines was reduced

Stress response in plants is an extremely complex physiological regulatory process. After being stimulated by external stress, plants can upregulate or downregulate the expression of miRNAs, causing changes in the expression levels of their target genes and leading to changes in metabolic products such as enzyme activity and osmoregulatory substances [40, 41]. When plants are subjected to stress, peroxidases related to reactive oxygen scavenging are produced, which can scavenge the accumulated ROS, thereby improving the plant’s resistance and enabling it to adapt to stress and maintain normal metabolism [42]. Consequently, to maintain normal plant physiology, the content of protective enzymes such as POD and SOD increase to scavenge hydrogen peroxides and free radicals [43]. In the present study, to analyse the molecular function of *Lol-miR11467* in larch, the physiological parameters of wild-type and overexpressing *Lol-miR11467* transgenic cell lines were determined to survey their ability to resist PEG_6000_ drought stress. Compared with the wild-type, the POD activity of the OE3 and OE12 was lower under PEG_6000_ stress (Fig. 3a), suggesting a decrease in antioxidant capacity. In addition, DEGs related to oxidoreductases, such as *POD*, were downregulated (Fig. 9b). Furthermore, although flavonoids can prevent the accumulation of ROS and exhibit a synergistic effect with antioxidant enzymes, such as POD, they can also resist the attack of ROS on the membrane system and reduce damage to plant cells [28]. However, in this study, the genes related to flavonoid synthesis were mostly downregulated (Fig. 8d). Therefore, overexpression of *Lol-miR11467* negatively regulated the expression of genes related to oxidoreductases such as POD and flavonoid synthesis, leading to a decrease in drought resistance in transgenic cell lines. The MDA content is used as an indicator of cell membranes damage by free radicals [44]. Compared with the wild-type, the content of MDA in the OE3 and OE12 was mostly higher, suggesting that the transgenic cell lines were more susceptible to membrane damage. The content of soluble protein first increased, then decreased, and finally increased again with the increase of stress time, which may regulate osmotic pressure by increasing the content of soluble protein after stress, thereby controlling water loss and avoiding damage. Compared to the POD and MDA content, the range of changes in soluble proteins was greater. The soluble protein content increased again after decreasing, possibly because of the induction of protein translation, such as stress proteins. Overall, compared with the wild-type, the content of soluble protein in transgenic cell lines was reduced. Previous studies showed that the increase and accumulation of soluble protein content can improve the water retention capacity of plant cells and protect cell life substances and biofilms. The higher the content, the stronger the ability to resist external stress [45]. Additionally, when plants are subjected to water stress, the osmotic regulation mechanism is activated, leading to a significant increase in soluble sugar content, thereby slowing down the damage suffered and enhancing plant’s drought resistance [46]. Moreover, the drought resistance of plants increases with the increase of soluble sugar content in plant cells [45]. In this study, soluble sugar content was lower in both OE3 and OE12 compared to the wild-type at different stress time (Fig. 3d). However, the soluble sugar content exhibited an increase within 48 h of stress and a subsequent decrease after 96 h. This suggests that in the short time, soluble sugars in larch callus tissue can serve as the main component osmotic regulatory substances to reduce cell osmotic potential and adapt to stress conditions. However, with the increase of PEG stress time, soluble sugar in callus tissue act as osmoregulatory substances to reduce water potential in plants, thereby reducing water leakage and maintaining normal metabolic activity. Similar studies were reported that soluble sugar content in *C.fissa* and *Helleborus orientlis* demonstrated an increasing and then decreasing, finally an increasing trend with overall with the drought strss [47, 48]. Furthermore, callus tissue growth increased under PEG_6000_ stress, possibly because of the low PEG_6000_ concentration and insufficient stress intensity. However, throughout the entire stress period, compared with the wild type, the increase in fresh weight of transgenic cell lines was much less than that of the wild type, indicating weakened drought resistance in transgenic cell lines, which was more unfavourable for their growth under PEG stress. The changes in POD activity, MDA, soluble protein and soluble sugar content, as well as callus growth after PEG_6000_ stress, demonstrated that the drought resistance of the overexpressing *Lol-miR11467* cell lines was reduced.

### Drought-responsive genes were downregulated in the overexpressing *Lol-miR11467* cell lines

MiRNAs mainly inhibit translation by splicing or incomplete pairing of target gene mRNA through complete pairing with the target gene, thereby completing their post transcriptional regulation in plants. Nearly two decades of research have revealed that each of these miRNA families targets a family of genes, with conserved miRNAs in plants corresponding to conserved target genes [49]. Many miRNAs respond to drought stress by regulating the expression levels of some genes, such as transcription factors, antioxidant defence mechanisms, signal transduction, and osmotic regulation [32]. Changes in transcription factor genes can induce the expression of downstream genes that alter plant stress tolerance. Previous studies have shown that miRNAs target genes related to drought stress include transcription factors SPL, ARF, MYB, TCP, GRAS, dehydroproteins, glutathione transfer, and ABA-related genes [42, 50]. AP2/EREBP is a transcription factor commonly found in plants, mainly involved in regulating responses to stress, such as high salt, drought, and hormones. Researchers have classified it into five major subgroups: AP2, RAV, DREB, ERF, and other categories [51]. In this study, to further investigate the regulation of target genes, 3147 common DEGs in transgenic cell lines were analysed, and many genes encoding transcription factors were downregulated, such as *bHLH*, *MYB*, *ERF*, and *WRKY* (Fig. 9a). Studies have shown that the overexpression of the *ERF* family transcription factor *OCRA3* gene can enhance plant resistance to salt stress. Overexpression of *OsERF71* in rice was reported to enhance drought tolerance [52]. Additionally, *WRKY* is upregulated in rice during drought stress and plays a significant role in rice’s drought resistance [53]. In this study, WRKY was downregulated in overexpressing *Lol-miR11467* cell lines, suggesting that *Lol-miR11467* negatively regulates the expression of *WRKY* genes, leading to a decrease in drought resistance in transgenic cell lines of larch.

In this study, many genes related to sugar metabolism and oxidoreductase were downregulated. Because sugar metabolism-related enzymes are related to osmotic regulation, an increase in these enzymes can enhance the permeability of plant cells, thereby resisting drought stress, and oxidoreductases are related to plant drought resistance [42]. Therefore, in this study, the downregulation of genes *UGT6*, *BGL1/8*, *GT*, and *SUS* may reduce the drought resistance of larch (Fig. 9c). Many genes with unknown functions (Table S14) were downregulated and could be aligned with *Picea sitchensis*. These genes may be specific to conifers and require further investigation. Simultaneously, *Lol-miR11467* suppressed the expression of many genes in larch, which was not conducive for adaptation to stress. In addition, other functional proteins genes, such as HSP, LEA proteins, and osmotic stress-related proteins, were also downregulated (Fig. 9d). Proteins that protect biomolecules in plants include LEA proteins, osmotic proteins, and chaperone proteins (HSP), and ubiquitin proteins Among them, the LEA protein is a widely existing protein related to osmotic regulation that accumulates under stress conditions, such as drought, salinity, and low temperatures [54]. Most osmotic proteins are stress proteins that are homologous to LEA proteins. Chaperone proteins are mostly HSPs, which can assist other macromolecules in folding and assembly, prevent protein denaturation, reduce cell water loss, protect membrane structure, and enhance plant adaptability to stress [55]. Previous studies showed a positive correlation between the LEA protein and stress tolerance. For example, overexpressing LEA in melon (*Cucumis melo* L.) can enhance tolerance to drought stress [21]. LEA was detected in the nutrient organs of wheat (*Triticum aestivum* L.) and maize (*Zea mays* L.) and played an important role under drought stress [55]. The above results indicated that overexpression of *Lol-miR11467* led to the downregulation of genes encoding these proteins; therefore, the osmotic potential of transgenic cell lines may be reduced due to their downregulation, causing a decrease in their drought resistance and ability to resist stress.

Based KEGG enrichment analysis, the phenylalanine biosynthesis pathway were significantly enriched in both OE3 vs. CK and OE12 vs. CK groups (Fig. 8a, b). Lignin can reduce water penetration and transpiration in plant cell wall, which can help maintain cell osmotic balance and protective membrane integrity [56, 57]. Previous studies indicated that the expression levels of lignin biosynthesis related genes (*COMT* and *CAD*) were significantly positively correlated with drought tolerance [58]. Similarly, compared with sensitive varieties, the expression levels of *PAL*, *C4H*, *4CL*, *CCR*, *CCoAOMT*, and *CAD* were unregulated in tolerant varieties under water stress, and overexpression of these genes enhanced lignin production, ultimately leading to increased drought tolerance in guar plants [59]. Over-expression of *MdPYL9* increased the drought resistance of plants by enhancing the up-regulated expression of the gene encoding 4CL [60]. In this study, lignin biosynthesis genes *PAL*, *4CL*, *COMT*, *CCoAOMT*, *CAD*, *CCR*, and *POD* were downregulated in both the OE3 vs. CK and OE12 vs. CK (Fig. 10). Additionaly, many studies showed that flavonoid metabolism is involved in plant response to drought stress [61, 62]. CHS and CHI are rate-limiting enzymes for flavonoid biosynthesis [63]. Coumaroyl-CoA is catalyzed by *CHS* to form chalcone [64]. Previous studies showed that upregulation of *CHS* genes improved the antioxidant capacity of A. *mongolicum* [65]. Overexpression of *CsF3H* promoted the biosynthesis of proanthocyanidins and salt stress resistance [66]. Overexpression of *ANR* can increase the content of proanthocyanidins and enhance stress resistance by scavenging reactive oxygen species [67]. Overexpression of *PuANR* can enhance drought resistance in *Populus ussuriensis* [68]. In our study, the transcription levels of *CHS*, *F3H*, *FLS*, *LAR*, *ANS* and *ANR* in the flavonoid biosynthesis pathway were mainly downregulated in the OE3 vs. CK and OE12 vs. CK (Fig. 10). Therefore, it is speculated that the drought resistance of transgenic cell lines may be reduced.

Studies on the reduction in resistance caused by the overexpression of miRNAs have been conducted in other species as well. Overexpressin*g* miR393 led to a decrease in the expression levels of the target genes *TIR1* and *AFB2*, resulting in decreased salt and drought resistance. Overexpression of *osa-MIR393* also led to a decrease in salt-alkali resistance in rice [69, 70], and similar results were observed in tomatoes as well [71]. Conversely, the knockdown of miR393 has been shown to improve drought tolerance [70]. Transgenic lines exhibited drought- and cold-resistance traits by silencing miR165/166 in *Arabidopsis thaliana* [57]. Therefore, plant stress resistance can be enhanced by silencing or inhibiting miRNA expression using the short tandem target mimic (STTM) method and by overexpressing their target genes [72, 73].

## Conclusions

In conclusion, the overexpression of *Lol-miR11467* reduced the calli growth and antioxidant enzyme activity under PEG stress. Genes associated with the phenylpropanoid metabolism, transcription factors, oxidoreductases and plant hormone signal transduction, and glucose metabolism were mainly downregulated in both OE3 and OE12 cell lines. These findings suggested that these genes may play key roles in the response of *Larix kaempferi* 3 × *L. gmelinii* 9 to PEG osmotic stress. Based on the findings, we speculated that the drought resistance of the overexpression of *Lol-miR11467* cell lines might be weakened. This study could provide a valuable reference for the molecular mechanisms of miRNAs and their impact on the drought resistance in larch, potentially contributing to the development of strategies for improving plant resilience to environmental stresses.

## Materials and methods

### Materials

The embryogenic calli of *Larix kaempferi* 3 × *L. gmelinii* 9 were induced and preserved through our previous studies [22], the immature cones of hybrid larch *Larix kaempferi* 3 × *L. gmelinii* 9 were induced on BM basic medium with 1.0 mg/L 2,4-D (2,4-dichlorophenoxyacetic acid) and 0.2 mg/L KT (kinetin). Hybrid larch has advantages and is superior to its parents mainly in growth, resistance, and wood properties. Studies indicated that *Larix kaempferi* × *L. gmelinii* has the characteristics of rapid growth, hard material, and strong disease and cold resistance [74]. In this study, the embryogenic calli of *Larix kaempferi* 3 × *L. gmelinii* 9 were used as the receptor materials for genetic transformation. The pCAMBIA1301 vector with hygromycin phosphotransferase hpt screening marker and *Agrobacterium tumefaciens* GV3101 strain were preserved in our laboratory. Media preparation and the cultivation were performed as described by Zhang [22].

### Construction of overexpression vector pCAMBIA1301-*Lol-miR11467*

#### Synthesis of target genes

The precursor sequence of *Lol-miR11467* was obtained by sRNA sequencing. Amplification primers for the target gene were designed (Table S1) using primer premier 6.0, and the amplification system and reaction were listed in Table S2. After the PCR, a 1% agarose gel was prepared and imaged using a gel imaging system. The gel containing the target gene band was quickly cut under ultraviolet light, transferred to a 2.0 mL centrifuge tube, and the target gene was recovered using a gel recovery kit (OMEGA, D2500, USA).

#### Construction of overexpression vector pCAMBIA1301-*Lol-miR11467*

*EcoR* Ӏ and *Hind* III enzyme digestion was used in the plasmid pCAMBIA1301 and the target gene *Lol-miR11467* gel recovery product. Agarose gel electrophoresis was used to detect enzyme digestion and recover the double-enzyme digestion product. The reaction system was shown in Table S3. The reaction procedure was as follows: enzyme digestion at 37℃ for 2.5 h, followed by enzyme inactivation at 65℃ for 20 min. The target gene *Lol-miR11467* fragment and the double enzyme cleavage product (mole ratio of 3:1–10:1) were mixed, the recombination reaction was performed at 37℃ for 20 min, and immediately transformed into DH5a. After the emergence of a single *E. coli* strain, positive clones were identified using PCR and sequencing. Successfully sequenced plasmids were extracted using a Plasma Mini Kit I (OMEGA, D6943-01, USA) and transformed into *Agrobacterium* GV3101, where positive colonies were detected. The primers used for detection were as follows: F: 5’-TGTGTGAGTTAGCTCACTCATTAGGC-3’, R: 5’-TGAAAATAAATGCATTATCACTTC-3’.

### Genetic transformation of larch

The embryogenic calli were selected from the proliferation medium and used as explants for Agrobacterium genetic transformation. The above positive colonies of Agrobacterium were selected and cultivated in LB liquid medium containing corresponding antibiotics, and then incubated at 28℃and 220 rpm for about 18 h until the OD_600_ was between 0.8 and 1.0; The bacterial cells were collected at 4℃, 8000 rpm for 15 min, resuspend the cells in 1/2 MS suspension to achieve an OD_600_ between 0.5, which was the infection solution. The embryogenic callus tissues were placed in the infection solution for 20 min, transfered them into co-culture medium for 2 days, and then sterilizated by 500 mg/L cefotaxime two times and sterile water three times, finally, screened and cultivated in BM medium containing 4 mg/L hygromycin and 200 mg/L cefotaxime.

### Detection of *Lol-miR11467* resistant calli

To detect whether the *Lol-miR11467* has been transferred into callus tissue, the DNA of wild-type, empty vector, and resistant calli cell lines (callus tissues were grown in antibiotic screening medium) was extracted. The PCR reaction system and procedure were listed in Table S4. To further detect the expression level of wild-type and transgenic calli, total RNA was extracted using a plant RNA extraction kit (BioTeke Corporation, RP3301, China). cDNAs were synthesized using a PrimeScript^™^ RT regent kit (Takara, Japan), and the real-time quantitative PCR (qRT-PCR) was performed on TransGen Biotech (Beijing, China) using ABI7500 (Applied Biosystems, USA). The primers used for detection in *Lol-miR11467* resistant calli were listed in Table S5. The qRT-PCR reaction procedure, system, and calculation method were with reference to Zhang [22].

### Morphological and biochemical changes of Transgenic callus tissue under PEG osmotic stress

Fresh wild-type and transgenic calli that had proliferated for 10 days were selected and cultured in proliferation medium (1/10-BM, as described by Zhang [22] containing 20% PEG_6000_ (prepared at a ratio of 1:20 g/mL). The growth status of callus tissues under PEG stress was observed. Three biological replicates per treatment were performed using three pieces of callus tissue. Each piece of callus tissue was freshly weighted to 0.5 g.

Furthermore, the fresh weight of wild-type and transgenic callus tissues was measured at 0, 12, 24, 48, and 96 h under PEG_6000_ stress, and then stored in a -80℃ refrigerator for the detemination of biochemical parameters. POD activity, MDA, soluble protein and soluble sugar content in transgenic calli and wild-type were determined by the corresponding kits (Comin, Suzhou, China) according to the manufacture instructions.

### RNA-seq analysis

In this study, the resistant callus tissues were selected as the experimental material, and three groups with nine samples sequencing materials were set up: (1) empty vector pCAMBIA1301 resistant callus tissue (CK), with three replicates; (2) pCAMBIA1301-*Lol-miR11467* resistant callus tissue OE3 with three replicates; (3) pCAMBIA1301-*Lol-miR11467* resistant callus tissue OE12 with three replicates. RNA-seq was analysed by bioacme (Wuhan, China) and the high-throughput Illunima HiSeqTM2000 sequencing platforms was used. The raw reads were filtered through Trimmomatic v0.36 software to ensue all sequences above 36 bp, and genome were assembled by Trinity software. Differentially expressed genes (DEGs) were screened using DESeq2, with the screening criteria set as log2|(Fold change)|>1 and *p*-value < 0.05. The functional annotation, heat map of DEGs were performed using TBtools software [75]. Additionally, the RNA-seq data were submitted to the Genome Sequence Archive (GSA) with submission number: CRA015326.

### Verification of RNA-seq data

Fifteen DEGs were randomly selected from the RNA-seq results, and cDNAs were synthesized using a reverse transcription kit (Takara, Japan). Subsequently, qRT-PCR was performed on TransGen Biotech (Beijing, China) using ABI7500 (Applied Biosystems, USA). The primers for qRT-PCR were listed in Table S6. The relative expression levels of these genes using the $\:{2}^{-\varDelta\:\varDelta\:\text{C}\text{t}}$method were determined with reference to Zhang [22].

## Electronic supplementary material

Below is the link to the electronic supplementary material.

Supplementary Material 1

Supplementary Material 2

Supplementary Material 3

Supplementary Material 4

Supplementary Material 5

Supplementary Material 6

Supplementary Material 7

Supplementary Material 8

Supplementary Material 9

Supplementary Material 10

Supplementary Material 11

Supplementary Material 12

Supplementary Material 13

Supplementary Material 14

Supplementary Material 15

Supplementary Material 16

Supplementary Material 17

Supplementary Material 18

Supplementary Material 19

## Data Availability

The RNA-seq data were submitted to the Genome Sequence Archive (GSA) with submission number CRA015326 and the accession number was PRJCA024255. The data presented in this study are available upon request from the corresponding author.
