# Supplementary figures and images for: Overexpression of Lol-miR11467 negatively affects osmotic resistance in Larix kaempferi 3 × L. gmelinii 9
Source: BMC Plant Biol. 2025 May 6;25:592. doi: 10.1186/s12870-025-06591-x (PMC12054245; doi:10.1186/s12870-025-06591-x)

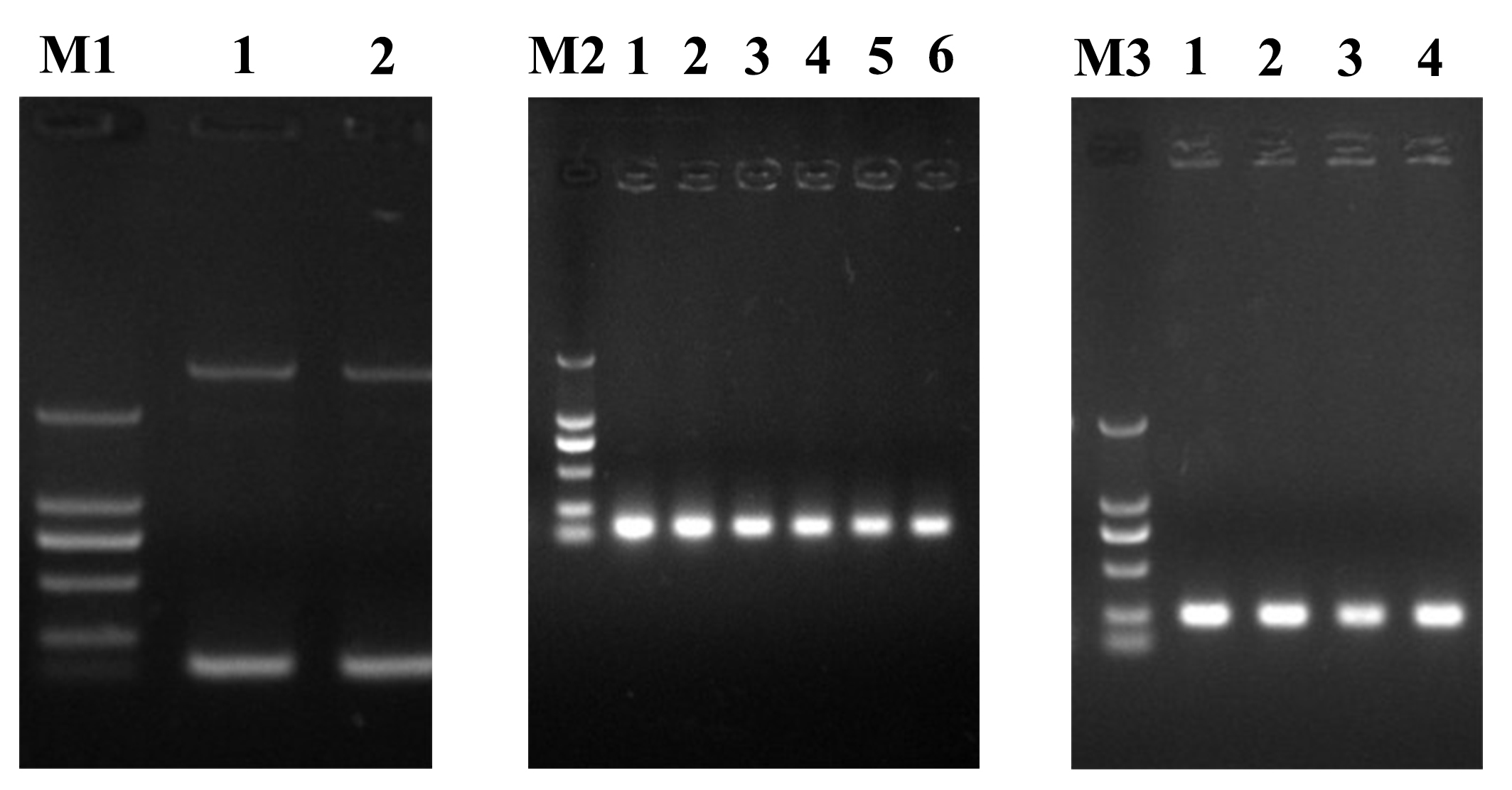

Supplement: Supplementary file 1 — Supplementary Material 1 [file 12870_2025_6591_MOESM1_ESM.jpg]

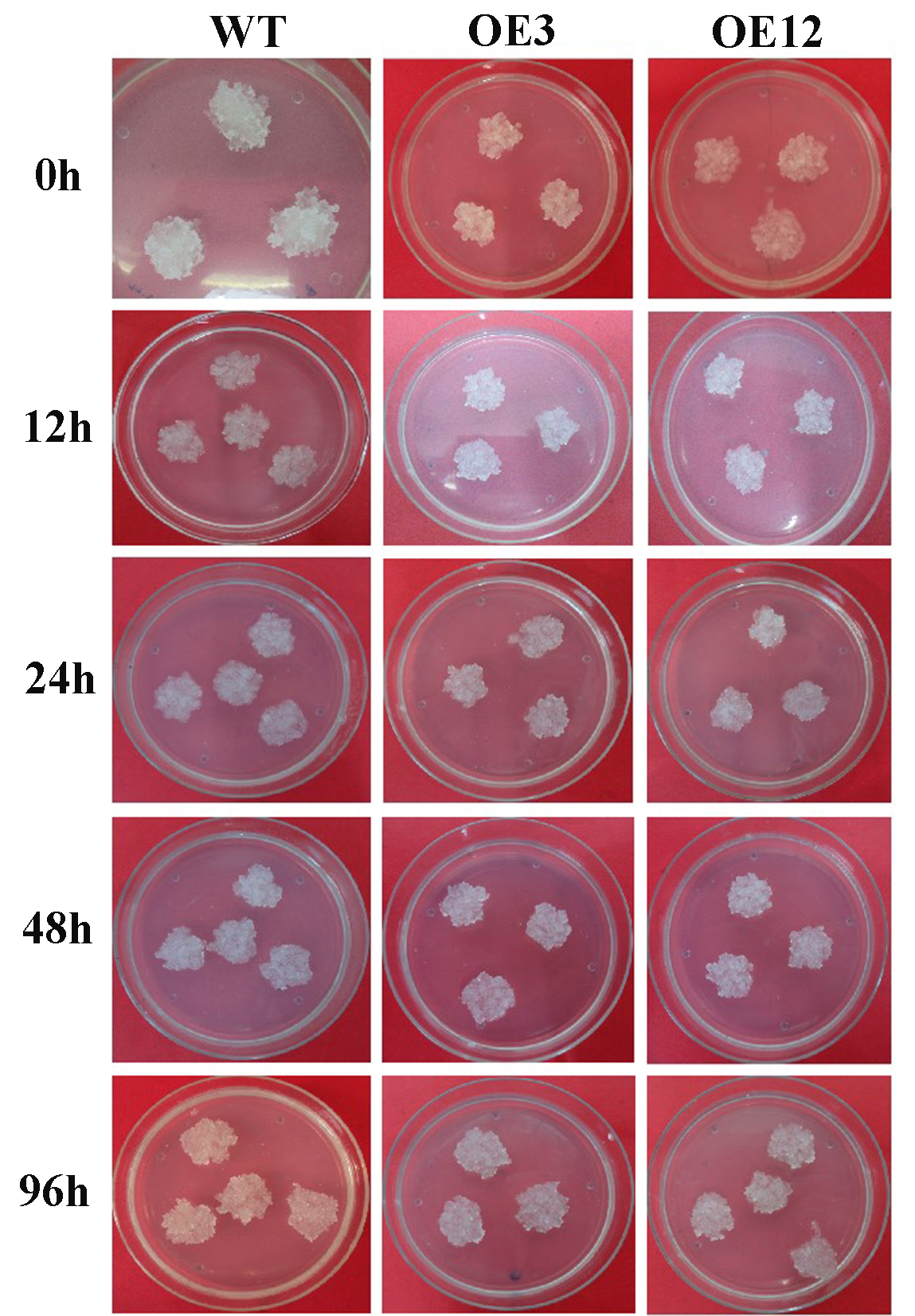

Supplement: Supplementary file 2 — Supplementary Material 2 [file 12870_2025_6591_MOESM2_ESM.jpg]

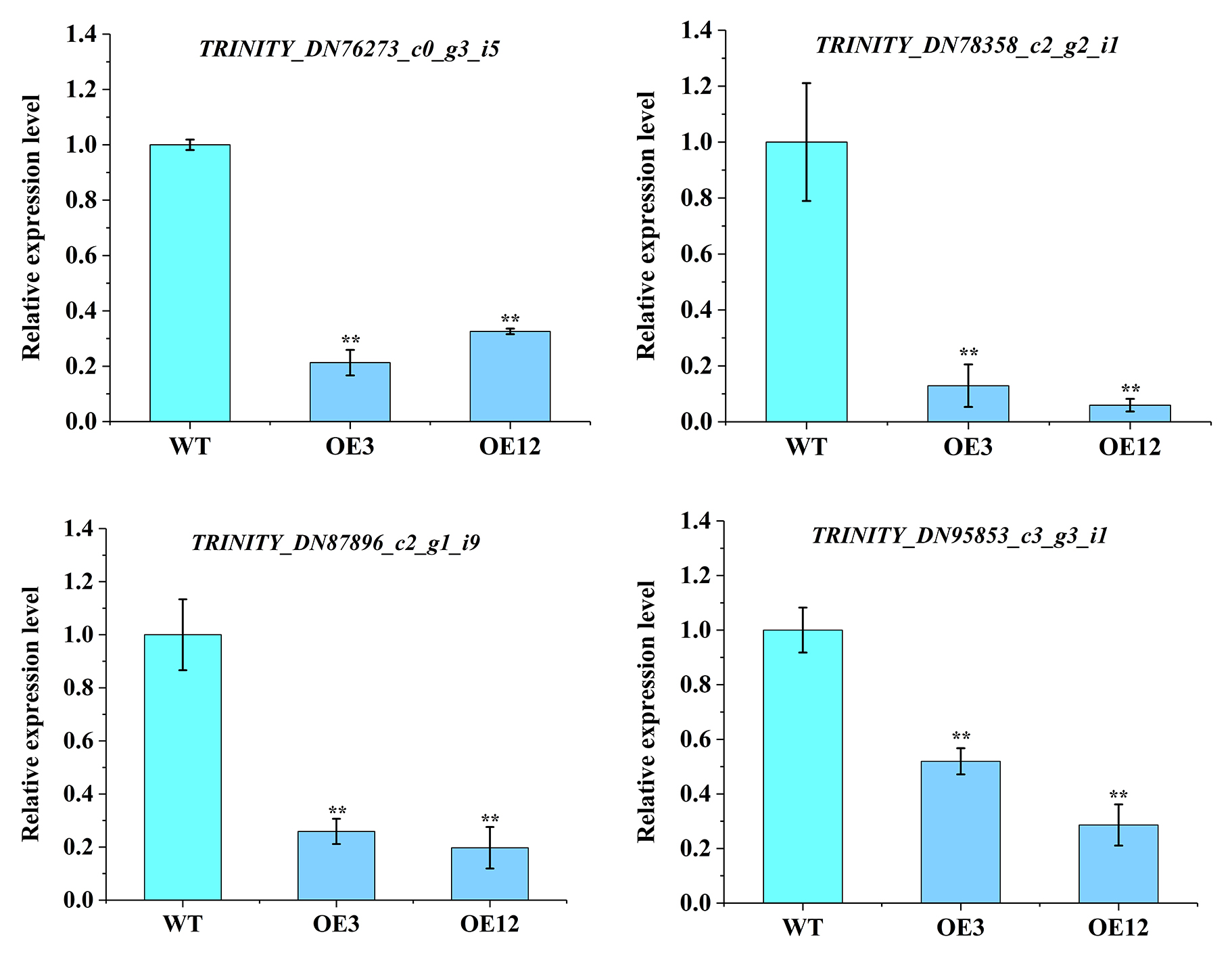

Supplement: Supplementary file 3 — Supplementary Material 3 [file 12870_2025_6591_MOESM3_ESM.jpg]
