# Supplementary material for: Overexpression of Lol-miR11467 negatively affects osmotic resistance in Larix kaempferi 3 × L. gmelinii 9
Source: BMC Plant Biol. 2025 May 6;25:592. doi: 10.1186/s12870-025-06591-x (PMC12054245; doi:10.1186/s12870-025-06591-x)
Supplement: Supplementary file 18 — Supplementary Material 18 [file 12870_2025_6591_MOESM18_ESM.pdf]

The following figures were the original, unprocessed versions of full-length gels and blots presented in the manuscript.

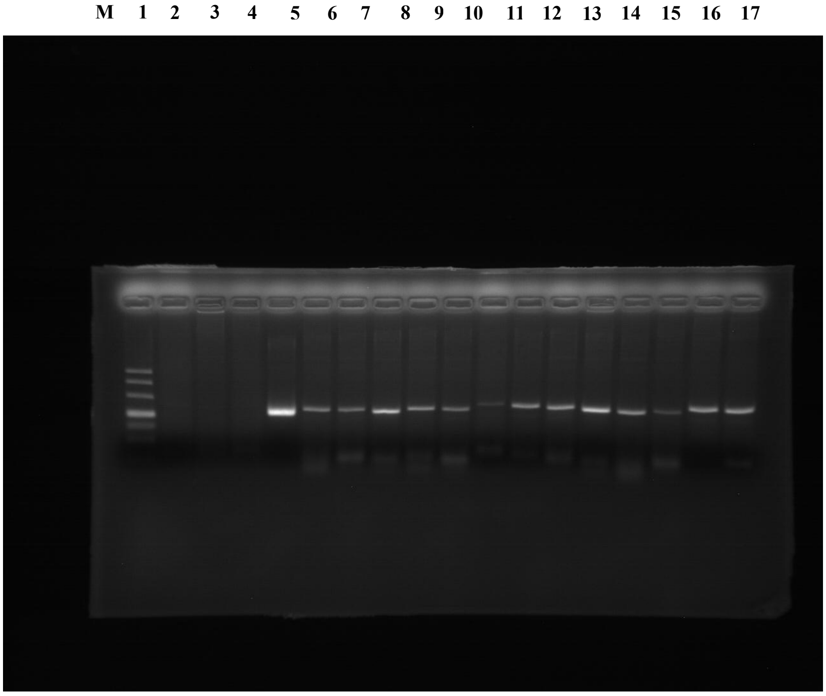

Figure 1 d. PCR detection of transgenic embryogenic calli. M: Marker DL500; 1-3: PCR product of water, wild-type and empty vector; 4: PCR product of pCAMBIA1301-*Lol-miR11467* plasmid; 5-17: PCR product of pCAMBIA1301-*Lol-miR11467* embryonic callus cell lines.

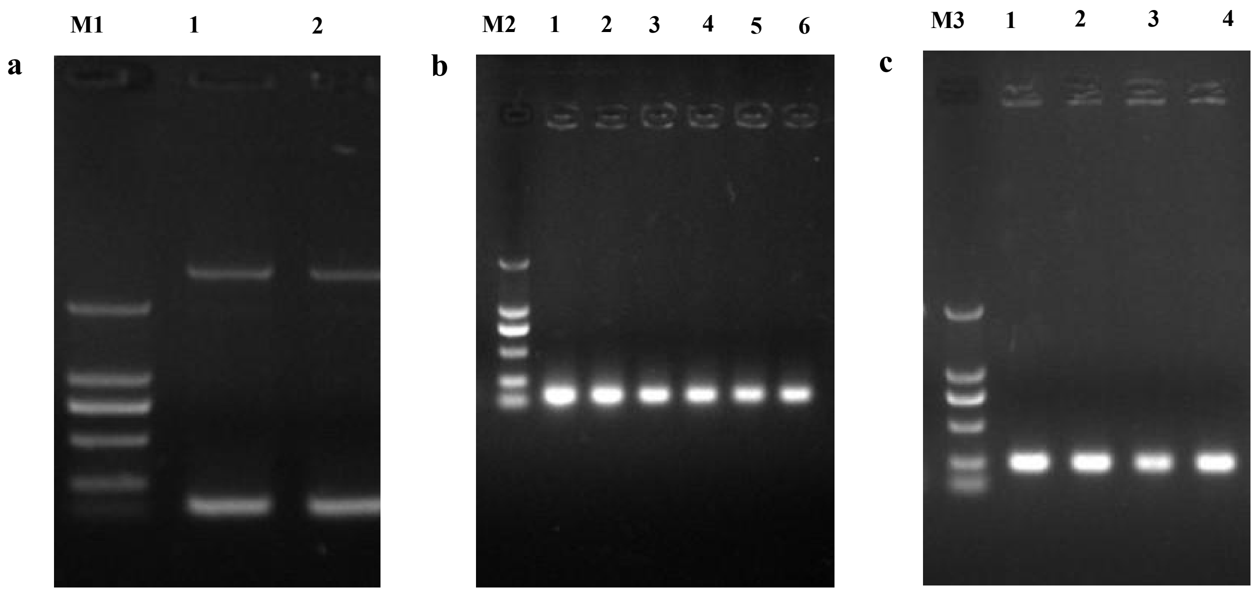

Figure S1. Vector construction. a. *Lol-miR11467* amplification electropherogram. M1 presents Marker DL2000, 1, 2 presents PCR product of *Lol-miR11467*; b. *E. coli* colony PCR detection of pCAMBIA1301-*Lol-miR11467*. M2 presents Marker DL2000; 1-6 presents E. coli colony PCR product of pCAMBIA1301-*Lol-miR11467*; c. Agrobacterium colony PCR detection of pCAMBIA1301-*Lol-miR11467*. M3: Marker DL2000; 1-4 presents *Agrobacterium* colony PCR product of pCAMBIA1301-*Lol-miR11467*.
