# Supplementary material for: Overexpression of Lol-miR11467 negatively affects osmotic resistance in Larix kaempferi 3 × L. gmelinii 9
Source: BMC Plant Biol. 2025 May 6;25:592. doi: 10.1186/s12870-025-06591-x (PMC12054245; doi:10.1186/s12870-025-06591-x)
Supplement: Supplementary file 19 — Supplementary Material 19 [file 12870_2025_6591_MOESM19_ESM.docx]

**Supplementary tables**

Table S1 Target gene amplification primer.

Table S2 Target gene PCR amplification system.

Table S3 Double digestion reaction system.

Table S4 PCR amplification reaction system.

Table S5 The primers used for detection of *Lol-miR11467* resistant calli.

Table S6 The primers used for quantitative real-time PCR.

Table S7 RNA-seq data sequencing quality assessment.

Table S8 Alignment analysis of clean data to the reference genome.

Table S9 Annotation of differentially down-regulated genes.

Table S10 The GO terms for enrichment of DEGs in OE3 vs CK and OE12 vs CK.

Table S11 The KEGG enrichment of DEGs in OE3 vs CK and OE12 vs CK.

Table S12 The prediction of target genes in *Lol-miR11467*.

Table S13 The primers used for qRT-PCR of predicted target genes in *Lol-miR11467*.

Table S14 Downregulated DEGs annotated as unknown function.

**Supplementary figures**

Figure S1. Vector construction. a. *Lol-miR11467* amplification electropherogram. M1 presents Marker DL2000, 1, 2 presents PCR product of *Lol-miR11467*; b. *E. coli* colony PCR detection of pCAMBIA1301-*Lol-miR11467*. M2 presents Marker DL2000; 1–6 presents *E. coli* colony PCR product of pCAMBIA1301-*Lol-miR11467*; c. Agrobacterium colony PCR detection of pCAMBIA1301-*Lol-miR11467*. M3: Marker DL2000; 1–4 presents Agrobacterium colony PCR product of pCAMBIA1301-*Lol-miR11467*.

Figure S2. Growth status of callus treated with PEG_6000_ stress at different time.

Figure S3. QRT-PCR analysis of predicted target genes in OE3 and OE12 transgenic cell lines.
